# SrO-Containing Bioactive Glasses in the SiO_2_–CaO–P_2_O_5_ System: A Comprehensive Review

**DOI:** 10.1021/acsbiomaterials.5c01097

**Published:** 2025-11-14

**Authors:** J. D. Salazar-Martinez, P. A. Forero-Sossa, J. Henao, A. L. Giraldo-Betancur

**Affiliations:** † Centro de Investigación y de Estudios Avanzados del Instituto Politécnico Nacional, 428213 Unidad Querétaro, Libramiento Norponiente, #2000 C.P. 76230 Querétaro, México; ‡ Tecnológico Nacional de México, 27744 Instituto Tecnológico de Querétaro, Av. Tecnológico S/N, Centro Histórico, Santiago de Querétaro 76000, QRO, México; § SECIHTI-CIATEQ A.C., Av. Manantiales 23-A, Parque Industrial Bernardo Quintana, El Marqués, Querétaro 76246, México

**Keywords:** bioactive glass, strontium oxide, melt-quenching, sol−gel, *in vitro* test, *in vivo* test

## Abstract

This comprehensive Review examines recent advances in bioactive glasses (BGs) based on the SiO_2_–CaO–P_2_O_5_–SrO system, highlighting the impact of the incorporation of strontium oxide (SrO) on their structural, thermal, and biological properties. By summarizing a wide range of studies, this work establishes how SrO modification influences the glass network connectivity, mechanical strength, and thermal stability, which are critical for high-temperature applications such as sintering and thermal spray coatings. This Review further explores the effects of SrO on *in vitro* and *in vivo* performance, focusing on the dissolution behavior, bioactive phase formation, cellular response, and antimicrobial activity. Special attention is given to the substitution and concentration level of SrO and their influence on bone regeneration and biocompatibility. This work offers insights into the design and optimization of Sr-doped BGs to enhance bone tissue engineering applications and lays the groundwork for future *in vivo* and clinical investigations.

## Graphical abstract

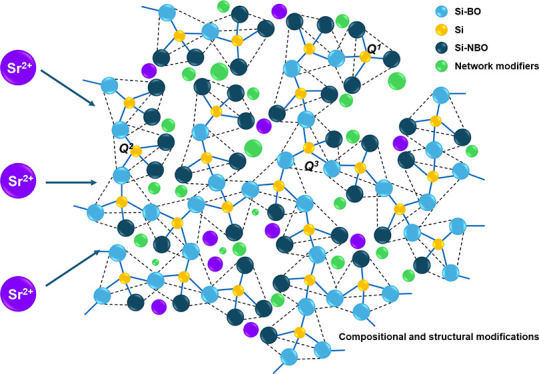

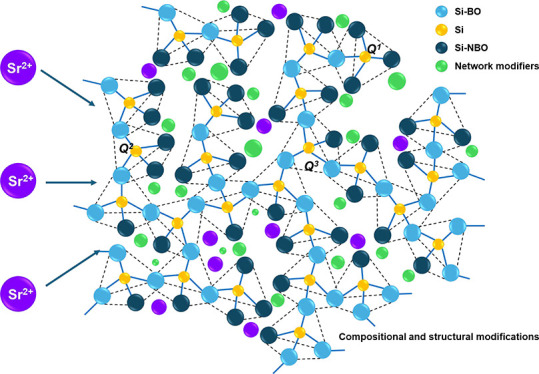

## 1 Introduction

Biomaterials are used in the biomedical field to treat, repair, and partially or totally replace tissues, organs, functions, and systems. To achieve this, they must have properties such as cytocompatibility, biodegradability, support for cell differentiation and proliferation, biocompatibility, and, in the case of applications related to the bone system, osteoinduction, osteoconduction, osteogenesis, and bioactivity, among others.^1^ The first generation of biomaterials emerged during the 1960s and 1970s. These materials were sought to be “bioinert” to avoid adverse reactions from the immune system and, at the same time, to exhibit a series of chemical, physical, and mechanical properties similar to those of the host tissue. Subsequently, between the 1980s and 1990s, the focus shifted toward materials capable of interacting with surrounding tissues and promoting biological responses at the tissue–material interface—these were termed “bioactive” materials, also known as biomaterials of second generation. From 2010 to the present, a new generation of smart biomaterials has been developed to mimic the hierarchical architecture of both soft and hard tissues, called third generation biomaterials. Among the most used biomaterials are polymers, metals, ceramics, bioactive glasses (BGs), and composites: materials composed of two or more constituents.^2^

BGs have been the subject of extensive research due to their potential applications in bone tissue engineering. However, their applications in soft tissue engineering have also been explored.^3^ The first BG composition was developed by Hench and colleagues in 1969 and was named “45S5 BG” (45SiO_2_-24.5CaO-6P_2_O_5_-24.5Na_2_O wt %). In 2000, this composition was approved by the FDA for its use in bone tissue applications.^4,5^ The 45S5 BG is currently employed in various commercial products intended for bone and dental treatment, including Bioglass, PerioGlass, and Ceravital, among others. After the disclosure of the 45S5 BG, numerous BG compositions have been developed over the past decades, with notable examples including ICIE16, S53P4, and 13–93.^6^ BG compositions are recognized for stimulating bone regeneration by releasing osteostimulatory dissolution products.^7^ The dissolution of BGs and the subsequent precipitation of bone-like apatite lead to the formation of similar structures and microstructures in both *in vitro* and *in vivo* environments.^8–10^ The dissolution behavior of BGs may occur homogeneously or heterogeneously and depends on surface characteristics as well as several factors, including glass composition, medium conditions, surface area to volume ratio, particle size, roughness, porosity, and temperature.^10^

The type of stimulation that occurs due to the dissolution of BGs can be adjusted by the incorporation of various ions at different concentrations.^11^ This customization is why BGs are considered third-generation biomaterials because they can be engineered to elicit targeted biological responses.^12^ In recent years, numerous studies (Figure 1) have focused on modifying the BG stoichiometry by incorporating ions capable of triggering specific physiological effects and exhibiting therapeutic properties. These trace elements, essential for numerous metabolic processes in the body, are commonly referred to as therapeutic inorganic ions (TIIs).^13^ As a result, they have been extensively studied and incorporated into a wide range of BG compositions. For instance, incorporation of sodium (Na^+^) into BGs enhances cytocompatibility and bioactivity. Similarly, adding magnesium (Mg^2+^) promotes cellular activity, apatite formation, angiogenesis, and improves mechanical performance.^14^ More recently, strontium ions (Sr^2+^) have gained considerable attention due to their positive impact on bone metabolism. Sr^2+^ can reduce bone resorption by inhibiting osteoclast activity and differentiation. Likewise, it can enhance the proliferation of preosteoblastic cells and promote osteoblast differentiation, thereby stimulating new bone formation. Moreover, Sr^2+^ has been shown to increase alkaline phosphatase (ALP) activity, collagen synthesis, and the expression of osteoblastic markers, thereby significantly enhancing osteogenesis.^14–16^ Additionally, Sr^2+^ also exhibits antibacterial activity against several strains, such as *E. coli*, *P. gingivalis*, and *S. aureus*, among others.^17^

**Figure 1:**
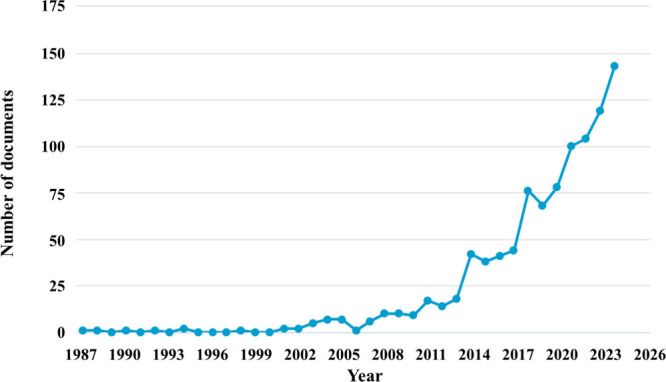
Number of publications published until November eighth, 2024, with the keywords “doped AND bioactive AND glass” on Scopus.

Several studies have investigated the synthesis and characterization of strontium-modified bioactive glasses (Sr-BGs). The amorphous network of these materials allows for the local and controlled release of Sr^2+^ ions at targeted bone sites. However, the incorporation of SrO alters the glass network, which may affect the thermal properties of the BGs in addition to their dissolution and ion release processes. These changes can subsequently influence the biological performance of the compositions both *in vitro* and *in vivo*. Since experimental results show differences depending on the SrO content and synthesis parameters, there is a need for a consolidated discussion of this information to guide further developments in the field. Such a comprehensive assessment can support the rational design of Sr-doped BGs with tailored properties for bone tissue engineering.

This Review focuses on summarizing the impact of SrO addition on the structure and properties of the bioactive SiO_2_–CaO–P_2_O_5_ glass system. It discusses various strategies for incorporating SrO into the glass network, highlighting structural changes at both atomic and macroscopic levels. Additionally, changes in thermal stability as a function of the SrO content and fabrication technique, bioactivity and overall biological performance are examined. Although the main focus of this Review is the incorporation of SrO into BG systems, some studies where SrO was replaced by other oxides are also considered. These works provide indirect but valuable evidence of SrO’s role by highlighting how its removal or substitution modifies glass properties. Finally, this Review offers insights into future research directions in this field.

## 2 Bioactive Glasses Based on Silicon in the SiO_2_–CaO–P_2_O_5_ System

BGs exhibit an amorphous structure with atoms distributed in a disordered manner. In some cases, they have short-range order (Figure 2a). The glassy structure is composed of network formers, modifiers, and intermediates. Network formers constitute the backbone of the glassy structure, and the most common are silicon oxide (SiO_2_), phosphorus pentoxide (P_2_O_5_), and boron trioxide (B_2_O_3_), whose characteristic units are SiO_4_, PO_4_, and BO_4_ tetrahedra, respectively. In the case of B_2_O_3_, BO_3_ units can also coexist with BO_4_ (Figure 2. Network modifiers disrupt the continuity of the network by introducing nonbridging oxygens (NBOs), which are not part of the tetrahedral network. They are usually alkali metal oxides and generate discontinuities in the glass network, reducing its network connectivity and increasing its tendency to crystallize. Network intermediates can act either as formers or modifiers depending on the overall composition. The structural disorder is further influenced by the flexibility of bond angles and variations in tetrahedral orientation. Additionally, interactions between modifiers and NBOs can induce local charge compensation, further altering the network connectivity. Depending on the alkali ion content, intermediates may shift their role, acting as modifiers in alkali-deficient systems or as formers when modifiers are abundant.^18–21^

**Figure 2:**
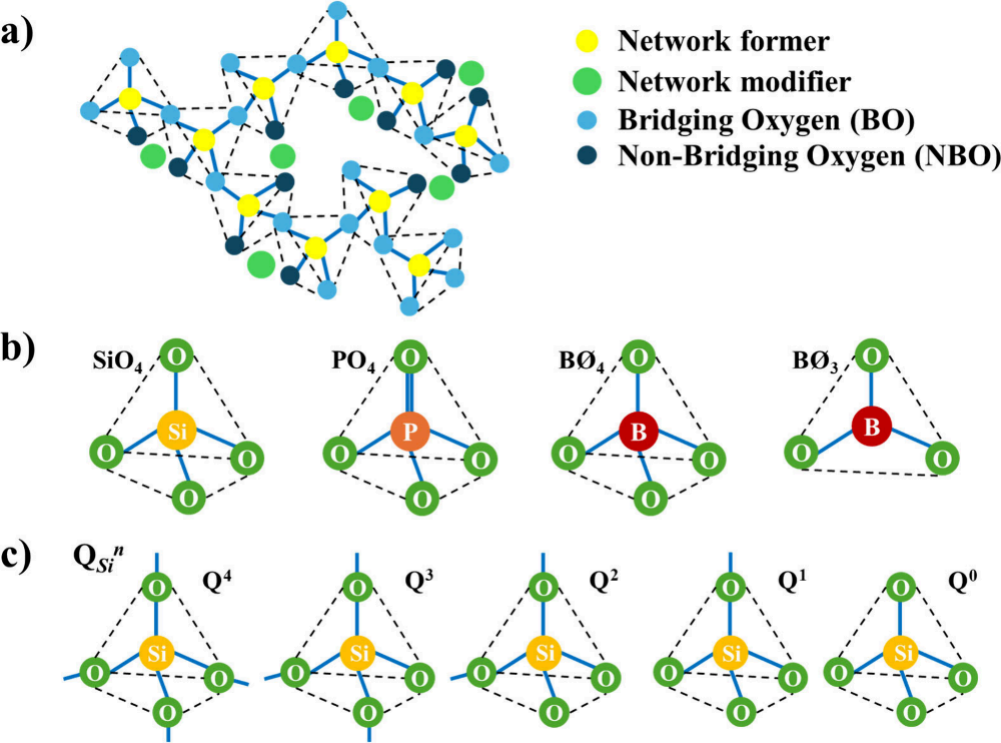
Schematic representation for (a) typical glass network, (b) common network formers, and (c) Q_Si_^*n*^ units.

Usually, BGs are given generic names based on their glass network formers. In the case of SiO_2_-based BGs, the leading glass network formers are SiO_4_ tetrahedral units connected by Si–O–Si- (Si-BO-Si) bonding oxygen (BO) bonds, and each BO is formed by two Si atoms. In contrast, each tetrahedron forms BOs with four adjacent SiO_4_ tetrahedra.^22^ These silicate tetrahedra and their associated bonds are represented as *Q*_Si_^*n*^ units, where *Q* are the Si ions bonded to *n* bonding oxygens (Figure 2c). The Si–O bonds are formed with angles between 120–180°. The angle, coupled with the length of the Si–O (∼0.162 nm) and the O–O (∼0.265 nm) bonds in the SiO_4_ tetrahedron, contributes to the randomness of the glass network.^20^ In some cases, oxygen defects may be present in the tetrahedral structure. However, when oxides from groups I and II of the periodic table are added, the oxygen-to-silicon ratio >2 for SiO_2_ generates oxygens with a single bond that do not participate in the glass network: monovalent cations need one oxygen, and divalent cations need two.^20^ Thus, these ions occupy interstices in the glass network and associate with NBO. Consequently, the formation of nonbridging oxygens (NBOs) leads to an increase in the coefficient of thermal expansion (CTE) of bioactive glasses.^20^

The BGs’ traditional manufacturing methods are sol–gel and melt-quenching.^23^ In general, the sol–gel method (Figure 3a) is a room-temperature chemical process that can obtain BGs through polycondensation reactions from organic and inorganic precursors, which have a high specific surface area and porosity, with pore sizes in the order of 2–50 nm. This process involves several stages: (i) preparation of precursor solutions at specific concentrations; (ii) mixing of the precursor solutions, leading to the gelatinization process; (iii) the aging process; (iv) removal of byproducts generated during the reactions using drying processes and the application of a final heat treatment.^24,25^ In the melt-quenching method (Figure 3b), precursor oxides are used in the quantities necessary to obtain the specific composition, which are mixed in a dry or humid medium and then melted at temperatures between 1300–1600 °C. The resulting molten liquid is rapidly cooled in graphite molds or water to avoid crystallization.^26^ However, in recent years, the use of other techniques such as spray pyrolysis,^27–29^ spray drying,^30–32^ the Evaporation Induced Self-Assembly (EISA) method,^33–35^ hydrothermal route,^36,37^ electrospinning,^38,39^ laser-spinning,^39,40^ and microwaves,^41,42^ among others, has been reported. Depending on the type of process used, BGs can be obtained with different particle sizes, morphologies, surface areas, and porosities, which in turn influence their performance and suitability for specific biomedical applications.

**Figure 3:**
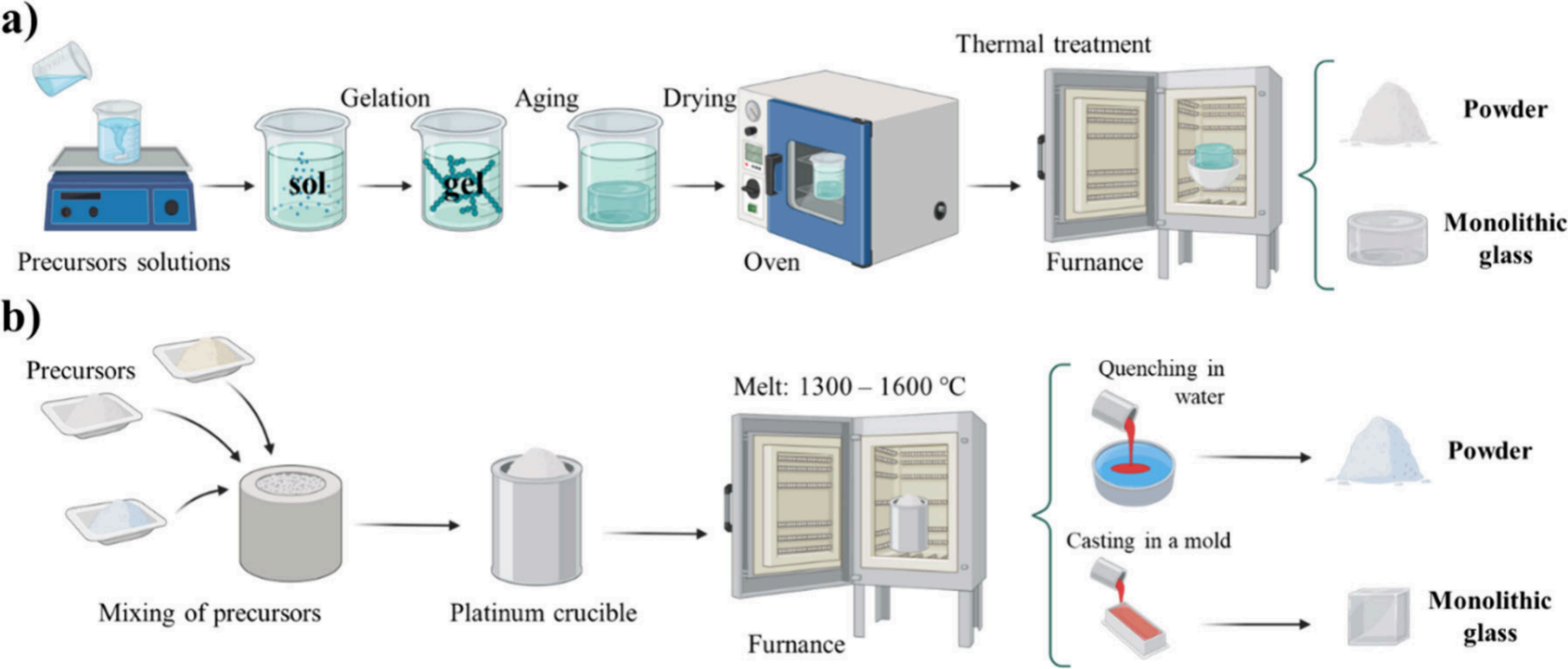
Traditional methods for BGs fabrication: (a) sol–gel and (b) melt-quenching (created with Biorender: https://app.biorender.com/illustrations/67290e49f6a2033104a768a7).

## 3 Degradation and Bioactivity Mechanisms

As a result of the interaction with body fluids, BGs can degrade in the body to give rise to new tissues, which is vital in implants aimed at repairing soft and hard tissue. Due to their amorphous structure, the dissolution processes of BGs can be controlled upon contact with physiological fluids. Their surface reactivity promotes ion exchange and precipitation reactions, resulting in the formation of a bone-like apatite layer. This layer establishes a strong physicochemical bond with the surrounding bone tissue and imparts osteoconductive properties to the BG. Furthermore, the ionic dissolution products can elicit various genetic responses related to osteogenesis, such as the stimulation of growth factor production, protein adsorption, and enhanced cell adhesion and proliferation.^43^ Thus, BGs that stimulate bone regeneration processes through dissolution products are considered osteostimulants.^7^ As reported elsewhere, the processes of BG dissolution and bone-like apatite precipitation generate similar structures and microstructures both *in vitro* and *in vivo.* ^8–10^

The dissolution processes of BGs can occur either homogeneously or heterogeneously and depend on the surface characteristics of the material and factors such as the composition of the BG, environmental conditions, the relationship between the surface area and the volume, particle sizes, roughness, porosity, and temperature, among others.^10^ According to the mechanism proposed by Hench and collaborators,^9^ the process of bone-like apatite formation is initiated by an exchange of ions, usually belonging to groups I and II of the periodic table, with protons (H^+^) from the physiological medium, generating an increase in the local pH. This pH increase promotes the breaking of -Si–O–Si- bonds of the BG and the subsequent release of Si ions into the physiological medium, leading to the formation of silanol groups (-Si–OH) (Figure 4a). Then, a layer of silica gel is generated due to the polymerization of the -Si–OH (Figure 4b), and in parallel, the release of Ca^2+^ and/or PO_4_^3–^ ions from the BG to the gel surface and/or into the physiological medium occurs. As the silica gel layer grows, microcracks may form due to surface tension, facilitating further ion release into the physiological medium (Figure 4c). The interaction between these released ions and the ionic species present in the medium (Ca^2+^, HPO_4_^2–^, PO_4_^3–^, and CO_3_^2–^, among others) promotes the nucleation of calcium phosphate (Figure 4d). Due to ionic interactions, the formation of an amorphous calcium phosphate (ACP) on the silica gel layer is favored (Figure 4e), and with further ion exchange, this ACP can react with ions present in the medium (OH^–^, Ca^2+^, Na^+^, HPO_4_^2–^, PO_4_^3–^, CO_3_^2–^, among others), promoting its transformation into a more crystalline bone-like apatite layer (Figure 4f).^44^

**Figure 4:**
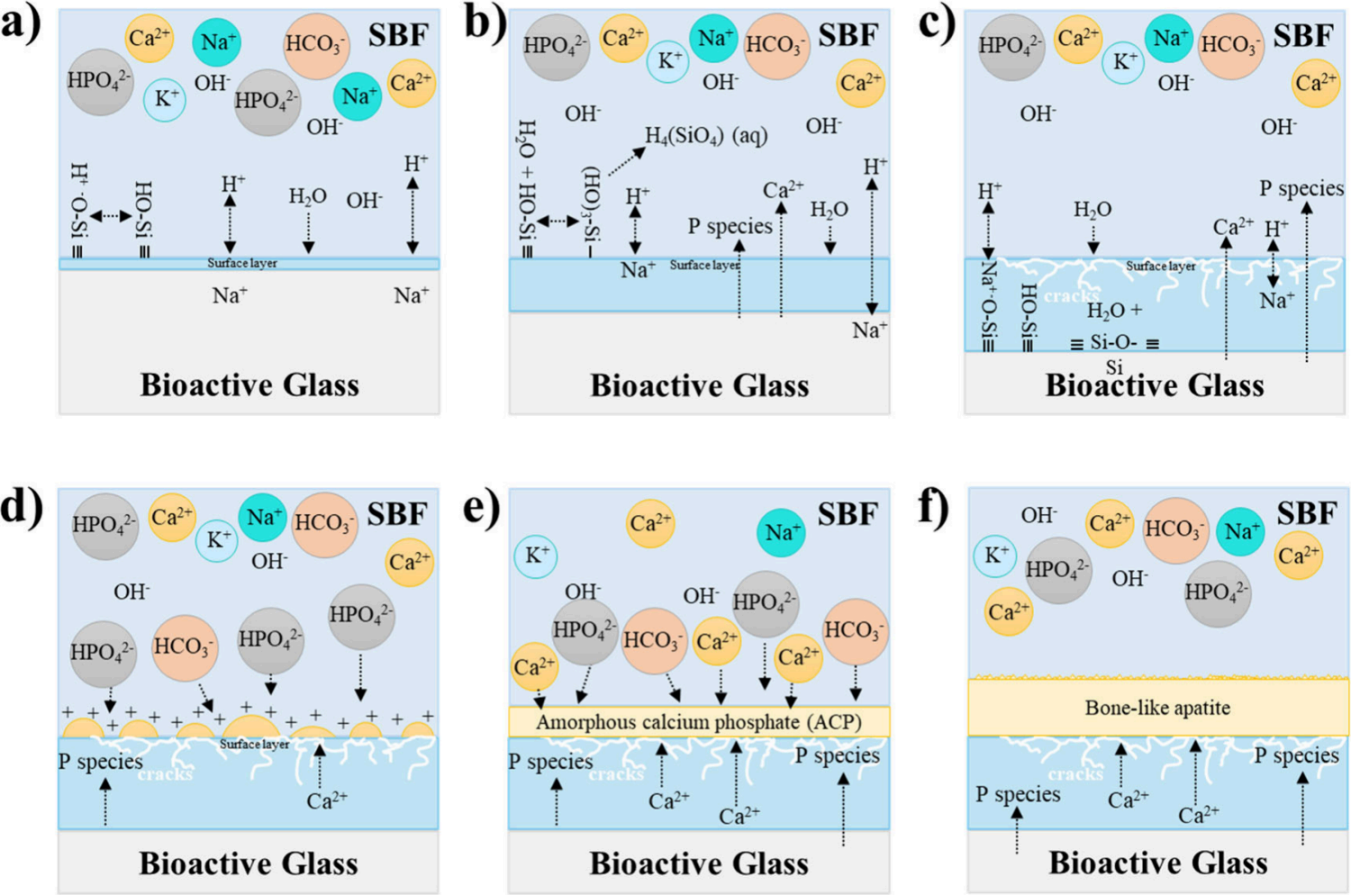
Bone-like apatite mechanism proposed for bioactive glasses in simulated body fluid (SBF) initiated by the silica gel formation (a, b) and the subsequent ion release (c), resulting in the nucleation, precipitation, and growing of the bone-like apatite layer (d–f).

## 4 Incorporation of Ions into the Glassy Network and Its Effects on the Biological Response

Owing to the network characteristics, BG compositions can be tailored with different TIIs to elicit specific biological responses upon their release in the human body (Figure 5).^11^ Thus, third-generation materials can be obtained by carefully controlling the type of ionic substitution and the concentrations in the glassy system.^12^ During this process, it is necessary to consider that these additions at different concentrations can affect the structure of the glassy network since the incorporated ions can act as network modifiers or intermediates and, therefore, affect the thermal parameters of the resulting compositions, modify the mechanical properties of the BGs, change the dissolution processes, the kinetics of ionic release, and the *in vitro* and *in vivo* performance of the compositions.^45–53^ Among the various ions, strontium (Sr^2+^) stands out due to its multiple physiological roles, making it highly relevant in the design of BGs.

**Figure 5:**
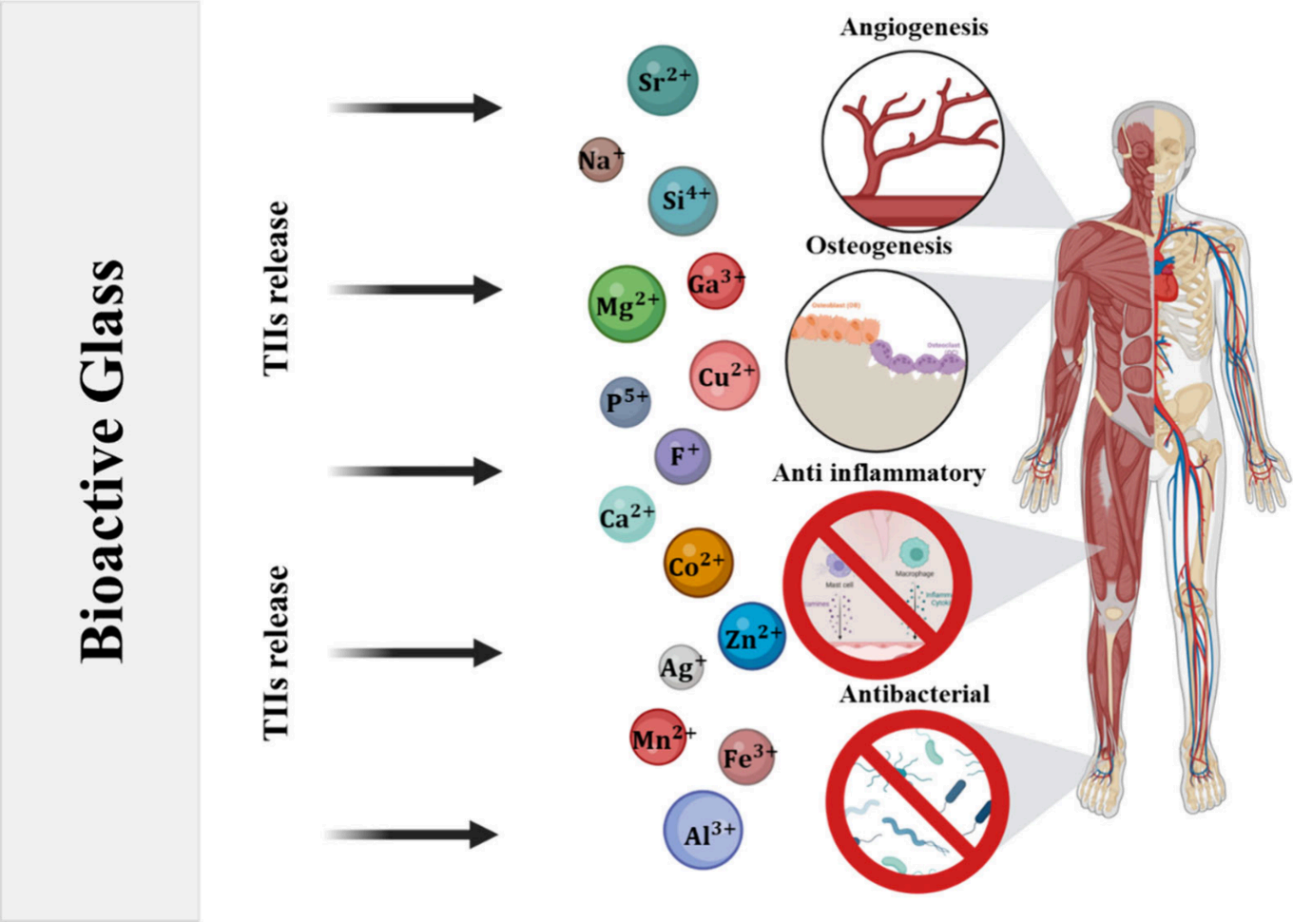
Schematic representation of some of the biological responses induced by therapeutic inorganic ions (TIIs) released by bioactive glasses (created with Biorender: https://app.biorender.com/illustrations/672aa131ed119849eab616f0).

### 4.1 Strontium (Sr)

Sr is an alkaline earth metal belonging to group II (2A) of the periodic table, along with Be, Mg, Ca, Ba, and Ra.^54,55^ The incorporation of Sr^2+^ into the bone mineral phase occurs primarily through passive substitution for Ca^2+^, favored by their similar ionic radii (Ca^2+^: 0.94 Å; Sr^2+^: 1.16 Å), rather than through active targeting or selective incorporation. The total Sr content in bone tissue is estimated at approximately 0.05 wt %,^56^ while its plasma concentration ranges from 0.11–0.31 μmol/L. In the human body, Sr can be retained in extracellular fluids and plasma, primarily through protein binding, as well as in both soft and hard tissues. It is excreted in ionic form via urine and feces.^57^ In recent decades, there has been increasing interest in the use of Sr^2+^ for the treatment of osteoporosis. The use of strontium salts, such as citrate, gluconate, or lactate, alongside calcium supplements has shown efficacy in improving bone density in patients with osteoporosis. Additionally, the radioisotope Sr-89 has been employed as an adjuvant in chemotherapy for bone cancer treatment due to its palliative effects on bone pain.^17^ Strontium ranelate (SrRan), an oral drug, has been used in osteoporotic treatments for postmenopausal patients.^58^ In 2014, the European Medicines Agency (EMA) restricted its use due to an increased risk of cardiovascular events in patients with a history of heart disease or circulatory disorders. However, the mechanism underlying these adverse effects remain unclear. Additionally, the oral bioavailability of Sr^2+^ has been reported to be less than 20%, underscoring the need for alternative strategies that enable localized and controlled release at specific treatment sites.^17,58^ Thus, the incorporation of Sr^2+^ into BGs has emerged as a promising strategy for treating osteoporotic fractures and related conditions.^59^ In this context, several studies have focused on the synthesis and evaluation of Sr-modified BGs (Sr-BGs). Due to their structure, BGs enable the targeted and sustained release of Sr^2+^ ions at the desired bone sites. However, variations in BG composition, the method of SrO incorporation, and the incorporation levels can induce structural changes within the glass network. These changes may impact the thermal characteristics of BGs, as well as the dissolution behavior, ionic release kinetics, and ultimately the *in vitro* and *in vivo* performance of the resulting compositions.

## 5 State of the Art: Incorporation of SrO into the SiO_2_–CaO–P_2_O_5_ System

This review focuses on the fabrication and evaluation of BG compositions within the SiO_2_–CaO–P_2_O_5_–SrO system, emphasizing the substitution strategies used to incorporate SrO, as well as the structural, mechanical, and thermal properties, alongside their *in vitro* and *in vivo* performance. Additionally, studies involving SrO-containing BGs incorporated into polymeric matrices were included, highlighting key findings related to their physicochemical and biological characteristics, to enrich the discussion. Each reference is specifically mentioned. Moreover, the compositions reported in the SiO_2_–CaO–P_2_O_5_–SrO system and other oxide contents <5 mol % were arranged in the ternary diagrams presented in Figure 6, where the P_2_O_5_ mol % was fixed. This discussion provides insights into the design and optimization of Sr-doped BGs for enhanced bone tissue engineering applications.

**Figure 6:**
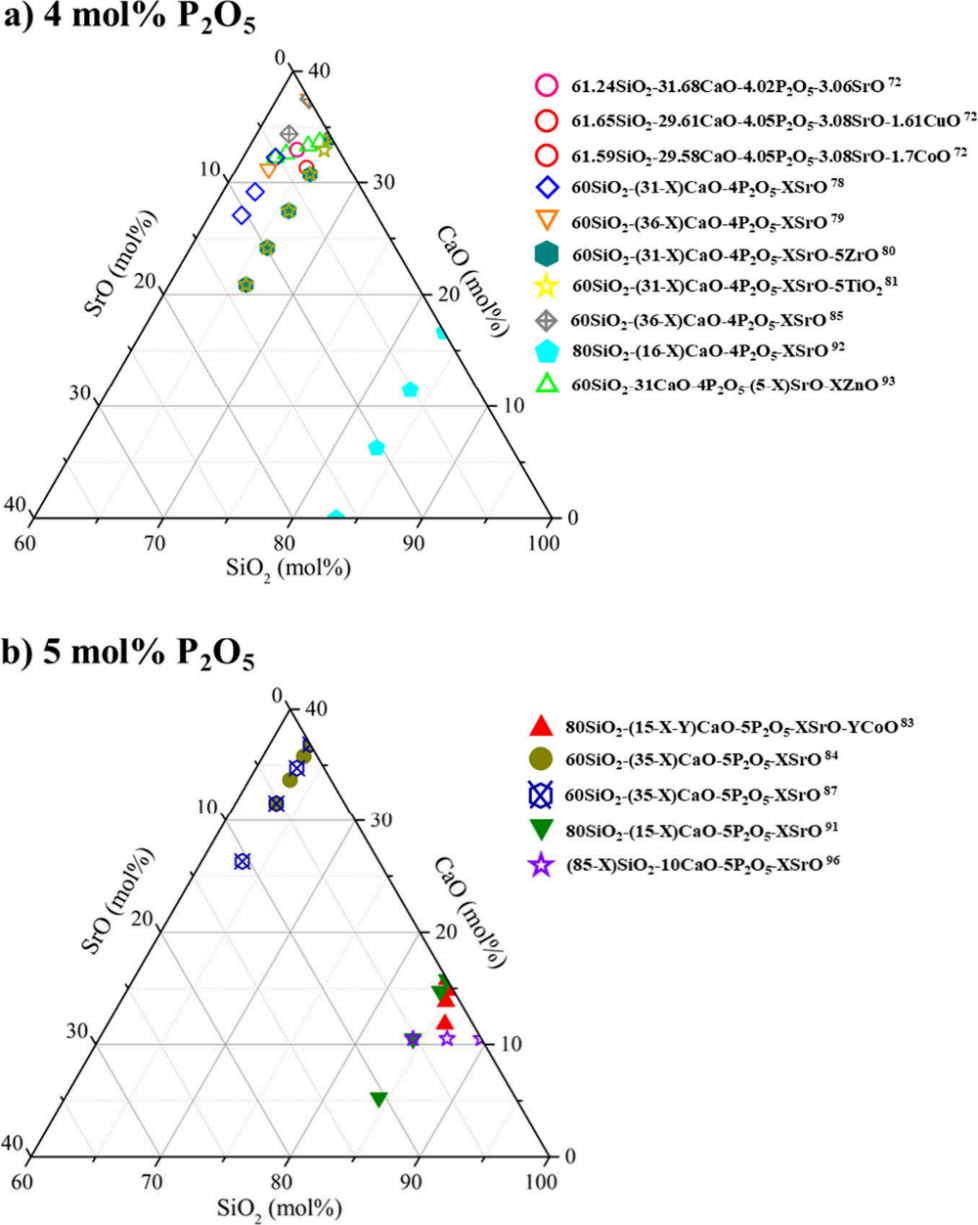
Ternary diagrams in the SiO_2_–CaO–SrO system for bioactive glasses formulated with fixed contents of (a) 4 mol % P_2_O_5_ and (b) 5 mol % P_2_O_5_ (compositions including additional oxides up to 5 mol % were considered within the systems).

### 5.1 Compositions with Fixed SrO Concentrations

Studies of compositions formulated with a specific percentage of SrO have been reported within the SiO_2_–CaO–P_2_O_5_ system. These investigations can be broadly divided into two categories: (i) those evaluating glasses in which the SrO content is fixed while other oxides are varied and (ii) those in which the SrO concentration itself is systematically modified. The first category is useful for understanding how SrO-containing glasses behave in multicomponent systems and how SrO may interact with other modifiers such as GeO_2_, ZnO, MgO, CuO, or CoO. However, in these studies, the actual role of SrO cannot be isolated, as the observed effects mainly derive from the oxides being varied while SrO remains constant. Table 1 summarizes different BG systems with fixed SrO content. By contrast, the second category provides more direct evidence regarding the influence of SrO concentration on the glass properties. Within the first category, Mokhtari et al. (2019)^60^ evaluated compositions in which SrO remained constant at 8 mol % and GeO_2_ content was systematically modified from the composition (48–X)­SiO_2_-6CaO-8SrO-36ZnO-2P_2_O_5_-XGeO_2_ mol % with X = 0, 6, and 12 mol % using the melt-quenching technique, which presented variations in the glass transition temperature (*T*_g_) values: 661, 668, and 653 °C for the compositions with X = 0, 6, and 12 mol %, respectively. The samples with X = 0 and 12 mol % presented a crystallization temperature (*T*_c_) at 856 and 840 °C, respectively, whereas the sample with X = 6 mol % exhibited two *T*_c_: the first at 868 °C (*T*_c1_) and the second at 935 °C (*T*_c2_). These *T*_g_ and *T*_c_ values are above those reported for the most reported BGs, such as 45S5 and S53P4, as shown in Figure 7. It was reported that the concentrations of PO_4_^3–^ and Zn^2+^ ions were less than 3 mg/L in all compositions after ion release tests in deionized water. A decrease in the concentration of Si^4+^ and Ca^2+^ ions, along with an increase in the concentration of released Sr^2+^ and Ge^4+^ ions, was observed as a function of the GeO_2_ content in the composites after 1000 h of immersion in deionized water. The authors associated these changes with the increase in NBOs in the glass network after the addition of GeO since they reported variations in the *Q*_Si_^*n*^ units: (i) an increase in the *Q*_Si_^0^ (14% and 22%) and *Q*_Si_^1^ (24% and 31%) units; (ii) a decrease in the *Q*_Si_^2^ (30% and 28%), *Q*_Si_^3^ (30% and 18%), and *Q*_Si_^4^ (2% and 0%) units in the composites with X = 0 and X = 12 mol %, respectively. However, these data were not presented for the sample with X = 6 mol %. Although SrO content was kept constant (8 mol %) in this system, the study is relevant because it highlights how the incorporation of GeO_2_, ZnO, MgO, CuO or CoO modifies the dissolution and structural behavior. These results cannot be attributed to SrO directly but provide insight into how SrO-containing systems behave in multicomponent networks.

**Figure 7:**
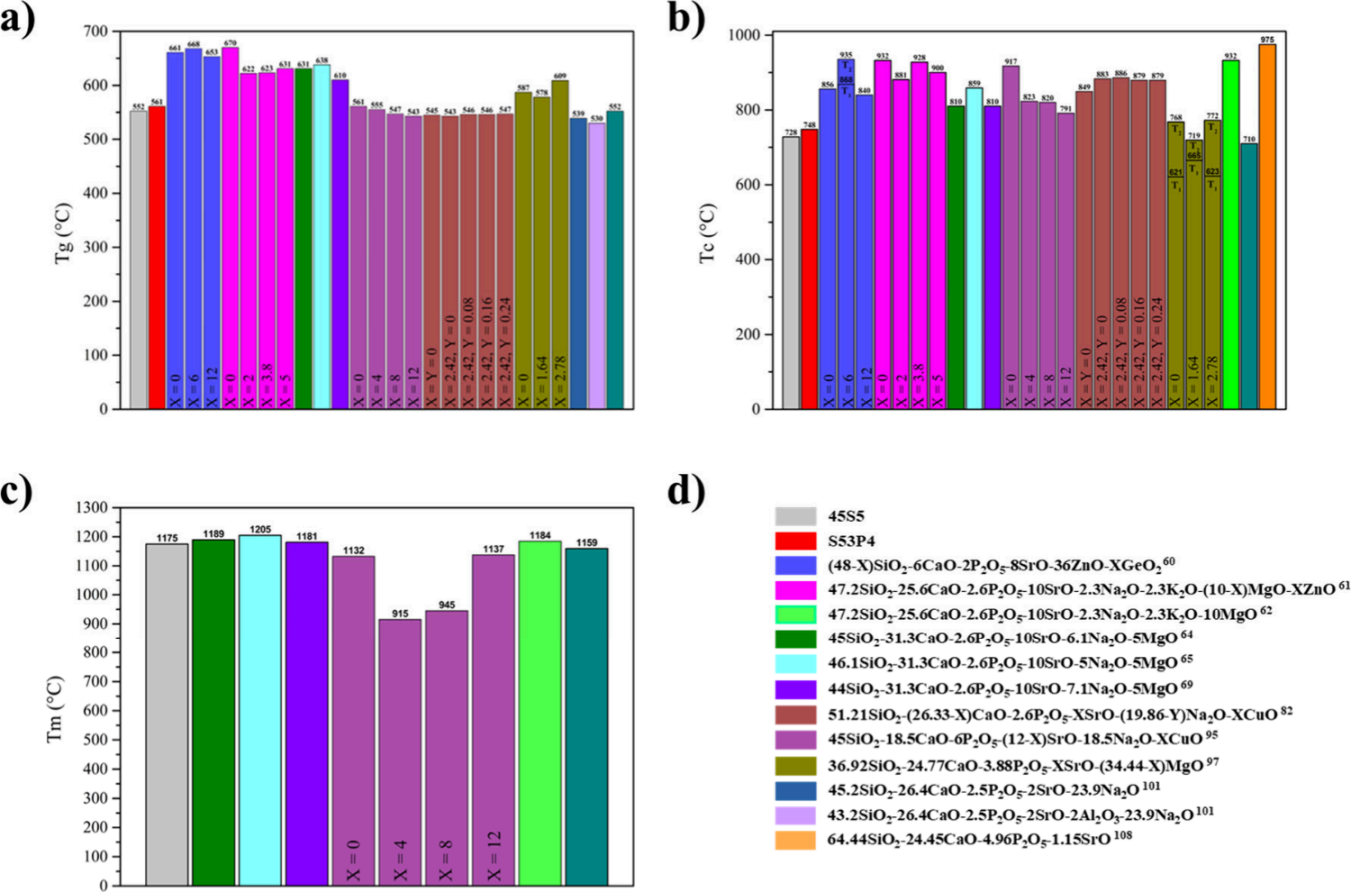
Values of (a) glass transition (*T*_g_), (b) crystallization (*T*_c_), and (c) melting temperatures (*T*_m_) for (d) some BGs formulations containing SrO reported in the literature compared with values reported for 45S5 and S53P4.

**Table 1:** Summary of BG Compositions Formulated with a Specific Percentage of SrO

glass composition (mol %)						*in vitro* bioactivity			cell assays		bacterial assays		
SiO_2_	CaO	P_2_O_5_	SrO	other oxides	structural features	solution	immersion times	bioactive phases	cell line	significant results	bacterial strain	significant results	ref
48	6	2	8	36 (ZnO)	amorphous	not reported			not reported		not reported		^60^
42	6	2	8	36 (ZnO), 6 (GeO_2_)	amorphous								
36	6	2	8	36 (ZnO), 12 (GeO_2_)	amorphous								
47.2	25.6	2.6	10	2.3 (Na_2_O), 2.3 (K_2_O), 10 (MgO)	amorphous	Kokubo’s SBF	1, 3, 7, and 14 days	not tested	MLO-Y4 (murine long bone osteocyte Y4) cells	not tested	*S. epidermidis*	no antibacterial activity	^61^
47.2	25.6	2.6	10	2.3 (Na_2_O), 2.3 (K_2_O), 8 (MgO), 2 (ZnO)	amorphous			not tested		cell viability		antibacterial activity	
47.2	25.6	2.6	10	2.3 (Na_2_O), 2.3 (K_2_O), 6.2 (MgO), 3.8 (ZnO)	amorphous			bone-like apatite formation		reduced cell viability		antibacterial activity	
47.2	25.6	2.6	10	2.3 (Na_2_O), 2.3 (K_2_O), 5 (MgO), 5 (ZnO)	amorphous			not tested		cytotoxicity		not tested	
47.2	25.6	2.6	10	2.3 (Na_2_O), 2.3 (K_2_O), 10 (MgO)	amorphous	Kokubo’s SBF	1, 3, and 7 days	bone-like apatite formation after 3 days of soaking	human bone marrow mesenchymal stem cells (hBM-MSC), L929 fibroblasts	adhesion, colonization, and cell differentiation	not reported		^62^
						Kokubo’s SBF	1, 7, and 14 days	bone-like apatite formation	human dental pulp stem cells (hDPSCs)	osteogenic activity	not reported		^63^
						not reported			MLO-Y4 (murine long bone osteocyte Y4) cells	cell proliferation	not reported		^73^
					amorphous structure in samples thermally treated at 650 and 750 °C	Kokubo’s SBF	3 and 7 days	bone-like apatite formation for sample with thermal treatment temperature of 750 °C	not reported		not reported		^70^
					amorphous matrix with crystalline contributions after thermal treatment of 850 °C			gel-silica layer					
					crystalline phases after thermal treatment of 950 °C			gel-silica layer					
45	31.3	2.6	10	6.1 (Na_2_O), 5 (MgO)	amorphous	Kokubo’s SBF	1, 3, and 7 days	bone-like apatite formation since 1 day of soaking	MLO-Y4 (murine long bone osteocyte Y4) cells	cell viability	not reported		^64^
46.1	31.3	2.6	10	5 (Na_2_O), 5 (MgO)	amorphous	Kokubo’s SBF	1, 3, and 7 days	bone-like apatite formation	L929 fibroblasts	cell viability	not reported		^65^
									human bone marrow mesenchymal stem cells (hBM-MSC)	osteogenic activity			
						not reported			MLO-Y4 (murine long bone osteocyte Y4) cells	cell proliferation	not reported		^73^
(42.16–34.99)	(18.82–39.98)	(14.87–11.52)	(7.13–2.21)	17.03–11.30 (Na_2_O)	not reported	Tris buffer solution (TBS)	not reported	bone-like apatite formation	not reported		*S. mutans*, *S. sobrinus*, *L. acidophilus*, and *L. rhamnosus*	antibacterial activity	^66^,^67^
53	15	4	5	23 (Na_2_O)	amorphous	Kokubo’s SBF	7 days	bone-like apatite formation	osteoblast-like MG-63 cells	cell proliferation and supported osteogenic differentiation	not reported		^68^
44	31.3	2.6	10	7.1 (Na_2_O), 5 (MgO)	not reported	Kokubo’s SBF	7 and 14 days	bone-like apatite formation	not reported		not reported		^69^
50	20	2	10	10 (MgO), 6 (ZnO), 2 (CuO)	amorphous and crystalline contributions	not reported			not reported		*E. faecalis* and *C. albicans*	antibacterial activity	^71^
50	20	2	15	5 (MgO), 6 (ZnO), 2 (CuO)	amorphous and crystalline contributions							antibacterial activity	
50	15	2	15	5 (MgO), 10 (ZnO), 3 (CuO)	amorphous and crystalline contributions							superior antibacterial activity against *E. faecalis*	
61.24	31.68	4.02	3.06		amorphous	Kokubo’s SBF	3, 7, and 14 days	calcite and bone-like apatite formation	rat-bone marrow mesenchymal stem cells (BM-MSCs)	cell proliferation and osteogenic effects	not reported		^72^
61.65	29.61	4.05	3.08	1.61 (CuO)	amorphous			calcite and bone-like apatite formation		increasing cell proliferation and osteogenic effects			
61.59	29.58	4.05	3.08	1.7 (CoO)	amorphous			calcite and bone-like apatite formation		increasing cell proliferation and osteogenic effects			

Similarly, Sergi et al. (2019)^61^ investigated glasses with a fixed SrO concentration (10 mol %), varying ZnO content to assess its influence on thermal behavior and *in vitro* performance of BGs obtained by melt-quenching, formulated from the composition BGMS10:2.3Na_2_O-2.3K_2_O-25.6CaO-(10-X)­MgO-10SrO-2.6P_2_O_5_-47.2SiO_2_-XZnO, with X = 0, 2, 3.8, and 5 mol %, previously developed by the research group. They reported *T*_g_ values of 670, 622, 623, and 631 °C for the samples with X = 0, 2, 3.8, and 5 mol % ZnO. *T*_c_ values of 932, 881, 928, and 900 °C were also presented for the compositions with X = 0, 2, 3.8, and 5 mol % ZnO, respectively. The authors proposed that the behavior could be associated with a Si–O–Zn bond strength lower than that of Si–O–Si. However, the *T*_g_ and *T*_c_ values reported in this study are higher than those of 45S5, S53P4, and others, as seen in Figure 7. After the *in vitro* tests, the precipitation of some morphologies associated with the presence of bone-like apatite was evidenced up to 14 days of immersion in Kokubo SBF in the sample with 3.8 mol % ZnO, which could be associated with a low ion exchange between the solution and this sample. Nevertheless, no results were reported for the other compositions. In cellular assays, a decrease in cell viability was observed with increasing ZnO in the compositions evaluated, such that the sample with 5 mol % ZnO promoted cytotoxic effects in MLO-Y4 osteocytes. Finally, it was determined that the compositions with 2 and 3.8 mol % ZnO inhibited bacterial growth against *S. epidermidis*. At the same time, they did not promote significant changes in bacterial growth against *E. coli* and *P. aueruginosa*. These effects could be related to the Zn^2+^ effect associated with the ZnO concentration and the dissolution processes of the samples. However, ionic release data were not provided for the evaluated compositions.

In another example with fixed SrO, Bellucci et al. (2019)^62^ studied the BGMS10:2.3Na_2_O-2.3K_2_O-25.6CaO-10MgO-10SrO-2.6P_2_O_5_-47.2SiO_2_ mol % composition containing 10 mol % SrO fabricated by melt-quenching, focusing on its in vitro bioactivity and cellular response induced by particles with sizes between 300–600 μm. As reported, bone-like apatite precipitation was observed after 3 days of immersion in Kokubo SBF. Cellular assays revealed that the particle extracts promoted cell viability in L929 fibroblasts as well as adhesion, colonization, and osteogenic differentiation of human mesenchymal stem cells (BM-MSCs) in the absence of exogenous growth factors. These effects were associated with ion release experienced by the BG in the experiments. However, no dissolution data were presented.

Following this line, Di Tinco et al. (2020)^63^ also examined the BGMS10 composition (10 mol % SrO fixed), reporting its osteogenic responses in Human Dental Pulp Stem Cells (hDPSCs), as indicated by the expression of the cellular markers STRO-1 and c-Kit, along with the expression of the FasL molecule, which is involved in the immunomodulatory functions of hDPSCs. Additionally, the activation of transcription factors RUNX2 and Osx, both linked to osteogenic differentiation, was identified. The authors suggested that these effects may be induced by ions released from the material; however, no data regarding the ionic release processes were provided. That same year, Sergi et al. (2020)^64^ developed the BGMSN glass: 45SiO_2_-31.3CaO-6.1Na_2_O-2.6P_2_O_5_-10SrO-5MgO mol %, synthesized via melt-quenching with a constant SrO content of 10 mol %, evaluating its thermal stability and *in vitro* performance. According to the study, this composition exhibited *T*_g_ = 631 °C, *T*_s_ = 711 °C, *T*_c_ = 851 °C, and *T*_m_ = 1189 °C, all higher than those reported for 45S5 and S53P4 (Figure 7). The coefficient of thermal expansion (CTE) was determined to be 8.87 × 10^–6^ (1/°C) over the 50–600 °C temperature range. The authors propose that the presence of Na_2_O, SrO, and MgO may influence the number of NBOs, thereby affecting the reported thermal parameters. However, no structural analysis of the glass network was presented. *In vitro* tests showed the formation of morphologies associated with bone-like apatite from the first day of immersion in Kokubo SBF, with increasing deposition up to 14 days. Additionally, this composition was found to support the viability of MLO-Y4 osteocytes. The authors suggested that the release of ions such as Sr^2+^ and Mg^2+^ may contribute to enhance the material’s biological response. Nonetheless, no data on ionic dissolution was provided.

Bellucci et al. (2020)^65^ reported the Bio_MS: 5Na_2_O-31.3CaO-5MgO-10SrO-2.6P_2_O_5_-46.1SiO_2_ mol % composition, synthesized by melt-quenching with 10 mol % SrO fixed, assessing its thermal properties and biological response. This composition exhibited *T*_g_ = 638 °C, *T*_s_ = 729 °C, *T*_c_ = 859 °C, *T*_m_ = 1205 °C, and Δ*T* = 179 °C, all higher than those reported for conventional BG compositions, as shown in Figure 7. The formation of a silica gel layer was observed after 3 days of immersion in Kokubo SBF, followed by the appearance of morphologies associated with bone-like apatite after 7 days. Cellular assays demonstrated that this composition promotes cell viability in L929 fibroblasts and stimulates osteogenic differentiation processes in BM-MSCs cultured without growth factors, effects that could be attributed to ions released from the BG. These promising results suggest potential for bone regeneration applications. However, no data on ionic release kinetics were provided, indicating the need for further investigation.

Dai et al. (2020)^66^ compared the antibacterial activity of the patented BG HX-BGC, formulated within the compositional range (42.16–34.99)­SiO_2_-(14.87–11.52)­P_2_O_5_-(18.82–39.98)­CaO-(17.03–11.30)­Na_2_O-(7.13–2.21)­SrO mol %, with that of the 3 M Specialty Glass 19933. The antibacterial performance was assessed against oral pathogens commonly implicated in oral caries including *S. mutans*, *S. sobrinus*, *L. acidophilus*, and *L. rhamnosus*. Results demonstrated that both BGs exhibited antibacterial properties, with HX-BGC showing significantly greater efficacy than BG 19933. Subsequently, Dai et al. (2021)^67^ evaluated the formation process of bioactive phases in Tris buffer solution (TBS) added with CaCl_2_ and K_2_HPO_4_ with different concentrations of the BG HX-BGC (1 and 5 mg/mL) and different concentrations of NaF (0, 1, and 5 ppm). In all conditions, the formation of bone-like apatite was observed. Notably, the simultaneous presence of Sr^2+^ ions from the BG and F^–^ ions from NaF favored the precipitation of strontium-substituted apatite or fluorine–strontium multisubstituted apatite, depending on the specific BG and NaF concentrations used.

Kumar et al. (2022)^68^ explicitly varied SrO concentration (5 mol % in this case) in mesoporous nanoparticles, providing direct evidence of SrO’s contribution to dissolution and biological effects. The BG nanoparticles with the composition 53SiO_2_-23Na_2_O-15CaO-4P_2_O_5_-5SrO mol % were prepared using the evaporation (EISA) technique, followed by a heat treatment at 600 °C for 3 h. The resulting material consisted of mesoporous, amorphous nanoparticles with an average size of approximately 10 nm and pore diameters of around 1.3 nm. *In vitro* tests revealed the formation of bone-like apatite after 7 days of immersion in Kokubo SBF. Additionally, extracts from the nanoparticles were found to enhance the proliferation of MG-63 osteoblasts like cells. The authors suggested that these biological responses could be related to the presence of SrO, the dissolution of BG, and the stimulation provided by the Sr^2+^ ions, as previously reported in the literature. However, no direct measurements of the ionic release from the samples were provided. It is noteworthy to mention that in this study, the bioactivity and cellular *in vitro* responses were not compared with a proper control BG without SrO. Thus, the *in vitro* performance of the material should be attributed to the combined effect of the ionic species (Si^4+^, Ca^2+^, P^5+^, Na^+^, Sr^2+^), rather than Sr^2+^ alone. Nonetheless, this report highlights the synergistic effects of SrO in combination with other modifiers instead of isolating its role.

Bellucci and Canillo (2022)^69^ developed the BGMS10_LS composition: 7.1Na_2_O-31.3CaO-5.0MgO-10.0SrO-2.6P_2_O_5_-44.0SiO_2_ mol % via melt-quenching with a fixed SrO concentration of 10 mol % to explore its thermal behavior and bioactivity. This glass exhibited characteristic temperatures of *T*_g_ = 610 °C, T_s_ = 686 °C, *T*_c_ = 810 °C, and *T*_m_ = 1181 °C (Figure 7). *In vitro* bioactivity assessments revealed the formation of bone-like apatite after 7 and 14 days of immersion in Kokubo SBF. It is important to note that the main compositional differences from their previous work were the increase in Na_2_O and the decrease in SiO_2_, in addition to the presence of SrO. Therefore, the observed biological and structural effects cannot be directly related to SrO alone but rather to the combined influence of Na_2_O, SrO, and MgO on the glass network. The authors hypothesized that these oxides may contribute to (i) modifications in the glass network, reflected in the reported characteristic temperatures, and (ii) variations in the dissolution behavior. However, no structural characterization or ion release data were provided to support these hypotheses.

Angioni et al. (2022)^70^ further processed the BGMS10 composition reported by Bellucci and Canillo^62^ (SrO fixed at 10 mol %) by spark plasma sintering, analyzing the effect of sintering temperature on crystallization and mechanical response, since a Tc = 932 °C was reported for this composition (as shown in Figure 7), making it attractive for its use in high temperature processes. The authors applied specific experimental conditions, heating the composition to T = 750, 850, 900, and 950 °C at a pressure of 16 MPa for 2 min. They reported a crystallization energy of 538.9 kJ/mol, which was much higher than the crystallization energies reported for the 45S5 (230–338 kJ/mol) and S53P4 (283–311 kJ/mol) compositions. While the sample consolidated at 750 °C exhibited an amorphous nature, the transition from amorphous to crystalline was observed from T = 850 °C: the presence of crystalline domains of Pseudowollastonite (α-CaSiO_3_) and wollastonite (β-CaSiO_3_) immersed in a glassy matrix was corroborated by TEM. In contrast, for temperatures of 900 and 950 °C, the presence α- CaSiO_3_ and β-CaSiO_3_ was observed as crystalline phases. Such results indicate that crystallization phenomena can occur at temperatures below the Tc peak (932 °C for this composition). Thus, an adequate interpretation of thermal data is recommended in order to select proper processing parameters in high temperature processes. Likewise, an increase in Young’s modulus values was determined as a function of temperature, which were 90.92 ± 3.42, 92.80 ± 2.22, and 98.38 ± 5.90 GPa for T = 750, 850, and 950 °C, respectively. Vickers’ hardness values of 671.7 ± 16.6, 718.8 ± 29.4, and 619.2 ± 47.1 HV were reported for T = 750, 850, and 950 °C, respectively. These variations are related to the structural features mentioned above: crystalline phases in the 850 and 950 °C consolidated samples. After 7 days of immersion in Kokubo SBF, bone-like apatite precipitation was observed in the sample sintered at 750 °C, and the presence of the silica gel layer was observed in the other samples tested, which could be associated with a slower apatite formation process for the samples sintered at 850 and 950 °C due to the presence of crystalline phases.

Correia et al. (2022)^71^ produced multicomponent glasses in the compositions: 50SiO_2_-20CaO-10MgO-10SrO-6ZnO-2CuO-2P_2_O_5_, 50SiO_2_-20CaO-5MgO-15SrO-6ZnO-2CuO-2P_2_O_5_, 50SiO_2_-15CaO-5MgO-15SrO-10ZnO-3CuO-2P_2_O_5_ by the *sol–gel* method and a heat treatment at 700 °C for 2 h, with fixed SrO concentrations (10–15 mol %) while simultaneously modifying MgO, ZnO, and CuO contents, highlighting the combined influence of these oxides. The results revealed amorphous phases alongside the formation of calcium strontium silicate (Sr_1.5_Ca_0.5_(SiO_4_)) in all of the fabricated samples. The author hypothesized that the presence and concentration of network modifiers, such as CaO, MgO, SrO, and CuO, could promote crystallization under the applied heat treatment conditions. Furthermore, the mesoporous nature of all compositions was corroborated. Additionally, it was observed that all of the compositions evaluated exhibited antibacterial activity against *C. albicans*. However, samples with higher SrO and ZnO contents exhibited greater antibacterial activity against *E. faecalis*. The authors also proposed that the antibacterial effects could be associated with the release of Si^4+^, Ca^2+^, Mg^2+^, P^5+^, Cu^2+^, Zn^2+^, and Sr^2+^ ions present in their samples. Still, no information was presented regarding the dissolution kinetics of the materials evaluated, limiting the understanding of the synergistic effect of SrO in combination with other modifiers such as CaO, ZnO, MgO, and CuO.

Alasvand et al. (2023)^72^ examined glasses with a fixed SrO content of ∼ 3 mol %, subsequently incorporating CuO or CoO (61.24SiO_2_-4.02P_2_O_5_-31.68CaO-3.06SrO mol % incorporating (i) CuO: 61.65SiO_2_-4.05P_2_O_5_-29.61CaO-3.08SrO-1.61CuO mol %, and (ii) CoO: 61.59SiO_2_-4.05P_2_O_5_-29.58CaO-3.08SrO-1.7CoO mol %) to study their combined effects on bioactivity and ion release as shown in Figure 6. These compositions were obtained by sol–gel, applying a subsequent heat treatment at 600 °C, with an unspecified treatment time. Amorphous materials with particle sizes <100 nm of mesoporous nature were obtained in all cases. Regarding the *in vitro* evaluations, bone-like apatite and calcite (CaCO_3_) formation was evidenced as bioactive phases in all samples after 14 days of immersion in Kokubo SBF. Ion release assays conducted in Kokubo’s SBF revealed higher concentrations of Sr^2+^ ions compared to the dopant ions Cu^2+^ and Co^2+^ in all doped samples, as expected. Moreover, the Sr^2+^ ion concentration was higher in the undoped composition than in the samples doped with CuO and CoO. These variations could be related to structural and bonding changes in the glass network, since copper, cobalt, and strontium partially substitute calcium positions. However, no release data were presented for the other elements in the samples, limiting the knowledge of the structural and dissolution phenomena caused by the synergistic effect of SrO, CuO, and CoO presence in the BGs. In addition, after tests with rat BM-MSCs, it was observed that all the evaluated samples promoted osteogenic processes but that this effect was greater for the compositions doped with CuO and CoO compared to the initial composition, which could be associated with (i) the concentrations of CuO and CoO used in the materials and (ii) the combined effect induced by Sr^2+^ and Cu^2+^, and Sr^2+^ and Co^2+^ ions in each sample. Nonetheless, the effect of ionic species such as Si^4+^, P^5+^ and Ca^2+^ should also be considered for an accurate interpretation of the mentioned findings.

Anesi et al. (2023)^73^ compared the previously reported BGMS10 ^62^ and Bio_MS^65^ compositions (each with 10 mol % SrO fixed) against 45S5, focusing on *in vitro* and *in vivo* biological performance with particle sizes between 100–500 μm. It was found that extracts of these samples at concentrations of 20, 40, and 60 mg/mL promoted cell proliferation in *in vitro* assays with MLO-Y4 osteocytes. In the *in vivo* assays, white New Zealand rabbits were used for 30 days (*n* = 12) and 60 days (*n* = 18), showing that all samples were biocompatible and osteoconductive. However, it was determined that 45S5 granules promote the formation of trabecular bone with greater density and, in turn, greater presence of soft tissues compared to BGMS10 and Bio_MS granules, which could be related to differences in the dissolution kinetics of the BGMS10 and Bio_MS compositions compared to that of 45S5.

Overall, the literature reports compositions with fixed SrO concentrations, generally ranging from 2–15 mol % in which additional modifiers are introduced to tune thermal, structural, and biological responses. In these systems, the outcomes—such as variations in *T*_g_, *T*_c_, bioactivity, and cytocompatibility—are primarily governed by the incorporated oxides (GeO_2_, ZnO, CuO, CoO, etc.), with SrO providing a constant background contribution. These works are valuable because they highlight potential synergistic effects of SrO in combination with other modifiers but do not isolate its role. On the other hand, a smaller set of studies has systematically varied SrO content itself within the SiO_2_–CaO–P_2_O_5_ system, providing clearer insight into how SrO concentration directly affects thermal stability, dissolution behavior, and biological performance. Taken together, these findings suggest that the presence of SrO is beneficial for bioactive glass performance, but they also underscore the need for further systematic studies focusing on the effect of different SrO concentrations on otherwise comparable compositions. The literature dedicated to such investigations is discussed in detail in the following sections.

### 5.2 Partially Substituted BGs from the SiO_2_–CaO-P_2_O_5_–SrO System

Due to the similarities between Ca^2+^ and Sr^2+^ ions, one of the alternatives explored for the incorporation of the latter into BGs of the SiO_2_–CaO–P_2_O_5_ system has been the substitution of CaO by SrO. However, depending on the initial composition of the BG and the substitution percentage, whether total or partial, different effects can be promoted both in the structure of the glass network and in the dissolution processes of the materials, which can have direct effects on the thermal and mechanical features and on the *in vitro* and *in vivo* performance of the resulting BGs. Thus, it is necessary to have a broader perspective regarding the percentage of substitution of CaO by SrO. The review of systems established in this section, including substitutions of 0–50% of CaO by SrO (summarized in Table 2) and 50–100% of CaO by SrO (summarized in Table 3), is a step toward inspiring new research and potential breakthroughs in this field.

**Table 2:** Reported BG Compositions where CaO is Substituted by SrO up to 50%

class composition (mol %)						*in vitro* bioactivity			cell assays		bacterial assays		
SiO_2_	CaO	P_2_O_5_	SrO	other oxides	structural features	solution	immersion times	bioactive phases	cell line	significant results	bacterial strain	significant results	ref
38.5	31	5.6	0	19.2 (MgO), 0.6 (CaF)	amorphous	not reported			human bone marrow mesenchymal stem cells (hBM-MSC)	cell proliferation	*S. aureus* and *E. coli*	antibacterial activity	^74^
38.5	31	5.6	0	15.2 (MgO), 0.6 (CaF), 4 (ZnO)	amorphous					cell proliferation		antibacterial activity	
38.5	31	5.6	0	13.2 (MgO), 0.6 (CaF), 6 (ZnO)	amorphous					cell proliferation		antibacterial activity	
38.5	27	5.6	4	15.2 (MgO), 0.6 (CaF), 4 (ZnO)	amorphous					cell proliferation		antibacterial activity	
38.5	27	5.6	4	13.2 (MgO), 0.6 (CaF), 6 (ZnO)	amorphous					superior cell proliferation		antibacterial activity	
38.5	25	5.6	6	19.2 (MgO), 0.6 (CaF)	amorphous					cell proliferation		antibacterial activity	
38.5	25	5.6	6	13.2 (MgO), 0.6 (CaF), 6 (ZnO)	amorphous					cell proliferation		superior antibacterial activity	
29.44	45.55	10.28	0	14.73 (MgO)	crystalline phases	Tas’ SBF	1, 7, and 14 days	bone-like apatite formation after 1 day of soaking	osteoblast-like MG-63 cells	enhancement in cell proliferation and viability with SrO content increasing	not reported		^75^
29.44	44.55	10.28	1	14.73 (MgO)	crystalline phases			bone-like apatite formation after 1 day of soaking					
29.44	42.55	10.28	3	14.73 (MgO)	crystalline phases			bone-like apatite formation after 1 day of soaking					
29.44	40.55	10.28	5	14.73 (MgO)	crystalline phases			bone-like apatite formation after 1 day of soaking					
41.2	36.14	5.06	0	7.17 (Na_2_O), 3.26 (MgO), 7.17 (K_2_O)	amorphous	Kokubo’s SBF	3 h, 24 h, 3 days and 7 days	bone-like apatite formation after 3 days of soaking	osteoblast-like MG-63 cells	cell viability	not reported		^76^
41.2	30.14	5.06	6	7.17 (Na_2_O), 3.26 (MgO), 7.17 (K_2_O)	amorphous			bone-like apatite formation after 3 h of soaking		cell viability			
41.2	35.14	5.06	0	7.17 (Na_2_O), 3.26 (MgO), 7.17 (K_2_O), 1 (CoO)	amorphous			bone-like apatite formation after 3 days of soaking		cell viability			
41.2	29.14	5.06	6	7.17 (Na_2_O), 3.26 (MgO), 7.17 (K_2_O), 1 (CoO)	amorphous			bone-like apatite formation after 3 h of soaking		cell viability			
53.85	22.65	1.72	0	21.77 (Na_2_O)	amorphous matrix with crystalline contributions	Hanks balanced salt solution	7 and 14 days	bone-like apatite formation	osteoblast-like MC3T3-E1 cells	cell proliferation and viability	*S. aureus* and *E. coli*	antibacterial activity	^77^
53.85	22.65	1.72	0	20.77 (Na_2_O), 1 (Ag_2_O)	amorphous matrix with crystalline contributions			bone-like apatite formation		cell proliferation and viability		superior antibacterial activity	
53.85	19.65	1.72	3	21.77 (Na_2_O)	crystalline phases			bone-like apatite formation		cell proliferation and viability		antibacterial activity	
53.85	19.65	1.72	3	20.77 (Na_2_O), 1 (Ag_2_O)	crystalline phases			bone-like apatite formation		superior cell proliferation and viability		superior antibacterial activity	
60	31	4	0	5 (MgO)	amorphous	Kokubo’s SBF	1, 3, 7, and 14 days	bone-like apatite formation	osteoblast-like G292 cells	not tested	not reported		^78^
60	28	4	0	8 (MgO)	amorphous			bone-like apatite formation		superior cell proliferation and viability			
60	26	4	0	10 (MgO)	amorphous			bone-like apatite formation		not tested			
60	31	4	5		amorphous			bone-like apatite formation		cell proliferation and viability			
60	28	4	8		amorphous			bone-like apatite and Ca_2_SiO_4_ formation		not tested			
60	26	4	10		amorphous			bone-like apatite, Ca_2_SiO_4_ and SrSiO_3_		not tested			
60	36	4	0		amorphous	not reported			macrophages RAW 264.7	low inhibition of osteoclast differentiation	not reported		^79^
60	30	4	6		amorphous					inhibition of osteoclast differentiation			
60	31	4	0	5 (ZrO_2_)	not reported	Kokubo’s SBF	3, 7, and 14 days	bone-like apatite formation after 3 days of soaking	osteoblast-like MC3T3-E1 cells	cell proliferation increases until X = 6 mol % SrO	methicillin-resistant *S. aureus* (MRSA)	antibacterial activity	^80^
60	28	4	3	5 (ZrO_2_)	not reported			bone-like apatite formation after 3 days of soaking				antibacterial activity	
60	25	4	6	5 (ZrO_2_)	not reported			bone-like apatite formation after 3 days of soaking				superior antibacterial activity	
60	22	4	9	5 (ZrO_2_)	not reported			reduction in formation rate of bone-like apatite		cell proliferation decreasing		antibacterial activity	
60	19	4	12	5 (ZrO_2_)	not reported			reduction in formation rate of bone-like apatite				antibacterial activity	
60	31	4	0	5 (TiO_2_)	amorphous	Kokubo’s SBF	1, 3, 7, and 14 days	bone-like apatite formation	osteoblast-like MC3T3-E1 cells	cell proliferation increases until X = 6 mol % SrO	Methicillin-resistant *Staphylococcus aureus* (MRSA)	superior antibacterial activity with SrO content increasing	^81^
60	30	4	1	5 (TiO_2_)	not reported			bone-like apatite formation					
60	28	4	3	5 (TiO_2_)	not reported			bone-like apatite formation					
60	25	4	6	5 (TiO_2_)	amorphous			bone-like apatite formation					
60	22	4	9	5 (TiO_2_)	not reported			bone-like apatite formation		cytotoxic effects			
60	19	4	12	5 (TiO_2_)	not reported			bone-like apatite formation					
51.21	26.33	2.6	0	19.86 (Na_2_O)	amorphous	not reported	not reported	not reported	not reported		not reported		^82^
51.21	23.91	2.6	2.42	19.86 (Na_2_O)	amorphous								
51.21	23.91	2.6	2.42	19.78 (Na_2_O), 0.08 (CuO)	amorphous								
51.21	23.91	2.6	2.42	19.70 (Na_2_O), 0.16 (CuO)	amorphous								
51.21	23.91	2.6	2.42	19.62 (Na_2_O), 0.24 (CuO)	amorphous								
80	15	5	0		amorphous	Kokubo’s SBF	4, 8, 24 h, 3 and 7 days	bone-like apatite formation after 8 h of soaking	osteoblast-like MG-63 cells	superior cell viability	*S. aureus* and *E. coli*	increasing antibacterial activity as a function of CuO content	^83^
80	14	5	0.5	0.5 (CuO)	amorphous			bone-like apatite formation after 8 h of soaking		decreasing cell viability with increasing CuO content			
80	13	5	1	1 (CuO)	amorphous			bone-like apatite formation after 7 days of soaking					
80	11	5	2	2 (CuO)	amorphous			bone-like apatite formation after 7 days of soaking					
60	35	5	0		amorphous	Kokubo’s SBF	7 days	bone-like apatite formation	osteoblast-like MC3T3-E1 cells	cell viability <70%	not reported		^84^
60	34	5	1		amorphous			bone-like apatite formation		increasing cell viability with SrO content			
60	32	5	3		amorphous			bone-like apatite formation		increasing cell viability with SrO content			
60	30	5	5		amorphous			bone-like apatite formation		increasing cell viability with SrO content			
60	36	4	0		amorphous	Kokubo’s SBF	1, 7, 14, and 21 days	calcite and bone-like apatite formation after 1 day of soaking	human osteosarcoma Saos-2 cell	cell viability	not reported		^85^
60	33	4	3		amorphous			calcite and bone-like apatite formation after 1 day of soaking		superior cell viability			
51.21	26.33	2.6	0	19.86 (Na_2_O)	amorphous	Kokubo’s SBF	7, 14, 21, and 28 days	bone-like apatite formation	not reported		not reported		^86^
51.21	23.91	2.6	2.42	19.86 (Na_2_O)	amorphous			superior bone-like apatite formation after 28 days of soaking					
51.21	23.91	2.6	2.42	19.78 (Na_2_O), 0.08 (CuO)	amorphous			reduced bone-like apatite formation with CuO increasing					
51.21	23.91	2.6	2.42	19.70 (Na_2_O), 0.16 (CuO)	amorphous								
51.21	23.91	2.6	2.42	19.62 (Na_2_O), 0.24 (CuO)	amorphous								
60	35	5	0		amorphous	Kokubo’s SBF	7 days	reduced bone-like apatite formation	not reported		*Streptococcus* spp. and *E. coli*	increasing antibacterial activity as a function of SrO content	^87^
60	33	5	2		amorphous								
60	30	5	5		amorphous			superior bone-like apatite formation					
60	25	5	10		amorphous								

**Table 3:** Designed BG Compositions by Substituting CaO by SrO above 50%

glass composition (mol %)						*in vitro* bioactivity			cell assays		bacterial assays		
SiO_2_	CaO	P_2_O_5_	SrO	other oxides	structural features	solution	immersion times	bioactive phases	cell line	significant results	bacterial strain	significant results	ref
46.1	26.9	2.6	0	24.4 (Na_2_O)	amorphous	not reported			not reported		not reported		^88^
46.1	0	2.6	26.9	24.4 (Na_2_O)	amorphous								
46.1	0	2.6	0	24.4 (Na_2_O), 26.9 (MgO)	amorphous								
35.5	45	6	0	7.5 (Na_2_O), 6 (CaF_2_)	amorphous	Tris buffer solution (TBS)	3, 6, 24 h, 3 and 7 days	not tested	not reported		not reported		^89^
35.5	45	6	0	7.5 (Na_2_O), 3 (CaF_2_), 3 (SrF_2_)				not tested					
35.5	45	6	0	7.5 (Na_2_O), 6 (SrF_2_)				fluoride substituted apatite formation after 6 h of soaking					
35.5	22.5	6	22.5	7.5 (Na_2_O), 6 (CaF_2_)	amorphous matrix with crystalline contributions			not tested					
35.5	22.5	6	22.5	7.5 (Na_2_O), 3 (CaF_2_), 3 (SrF_2_)				increasing of crystalline peaks					
35.5	22.5	6	22.5	7.5 (Na_2_O), 6 (SrF_2_)				not tested					
35.5	0	6	45	7.5 (Na_2_O), 6 (CaF_2_)	crystalline matrix with amorphous contribution			not tested					
35.5	0	6	45	7.5 (Na_2_O), 3 (CaF_2_), 3 (SrF_2_)				increasing of crystalline peaks					
35.5	0	6	45	7.5 (Na_2_O), 6 (SrF_2_)				not tested					
80	10	10	0		amorphous	Kokubo’s SBF	1, 3, 7, and 14 days	bone-like apatite formation	Wharton’s Jelly-derived mesenchymal stem cells (WJ-MSCs)	cell proliferation	not reported		^90^
80	0	10	10		amorphous matrix with crystalline contributions			strontium apatite formation with superior bioactivity performance		cell proliferation			
80	15	5	0		amorphous	Kokubo’s SBF	4, 8, 24, 72, 168, and 336 h	bone-like apatite formation	human bone marrow stem cells (hBMSC)	increasing in cell activity up to 5 mol % SrO	not reported		^91^
80	14	5	1		amorphous			strontium substituted bone-like apatite formation					
80	10	5	5		amorphous			strontium-substituted bone-like apatite formation					
80	5	5	10		amorphous matrix with crystalline contributions			strontium-substituted bone-like apatite formation		lower cell activity			
80	16	4	0		amorphous matrix with crystalline contributions	Kokubo’s SBF	14 days	bone-like apatite formation	bone marrow mesenchymal stem cells (BMMSCs) extracted from adult rats	osteogenic potential	not reported		^92^
80	11	4	5		amorphous matrix with crystalline contributions			bone-like apatite formation		osteogenic potential			
80	6	4	10		amorphous matrix with crystalline contributions			strontium apatite and strontium carbonate formation		superior osteogenic potential			
80	0	4	16		amorphous matrix with crystalline contributions			strontium apatite and strontium carbonate formation		osteogenic potential			

Regarding to substitutions of 0–50%, Popa et al. (2019)^74^ evaluated the effect of simultaneous addition of ZnO and SrO on the performance of BGs fabricated by the melt-quenching method from the system: 38.5SiO_2_-(36.1–Y)­CaO-5.6P_2_O_5_-(19.2–X)­MgO-0.6CaF_2_-XZnO-YSrO mol %, with (i) Y = 0, X = 0, 4, 6; (ii) Y = 4, X = 4, 6; (iii) Y = 6, X = 0, 6 mol %. The results showed that increasing both dopants led to a rise in the coefficient of thermal expansion (CTE). The composition with Y = 6, X = 6 mol % exhibited a CTE value (∼8.8 × 10^–6^ °C^1–^) very close to the values reported for Ti and its alloys (∼ 8.5–9.6 × 10^–6^ °C^1–^), suggesting its potential use in high-temperature processing applications. It was also shown that, with the increase in dopants, the amount of NBOs in the compositions is increased, as evidenced by variations in Raman bands intensities. However, no quantitative data regarding *Q*_Si_^*n*^ units were presented by the authors. Ionic release tests in the culture medium revealed varying behaviors across compositions. Particularly, in the case of P^5+^, similar concentrations were observed in all the compositions evaluated. In the case of Zn^2+^ ions, an increase in ionic release was evident as a function of the ZnO content in the samples, as expected. However, this trend was not observed for the release of Sr^2+^ with an increasing SrO content. In addition, there were variations in the concentrations of released ions, with no clear correlation with the SrO and ZnO content, since in the composition with X = 0, Y = 6 mol %, a greater release of Ca^2+^, Sr^2+^, and Mg^2+^ ions and a lower release of Si^4+^ was observed compared to the other doped samples. These findings suggest that multiple additions of ZnO and SrO cause several structural and bonding modifications within the glass network. Despite the charges of ionic species being the same (Ca^2+^ by Sr^2+^ and Mg^2+^ by Zn^2+^), the differences in ionic radii can cause significant changes in the connectivity of the glass network that will affect the dissolution behavior of the resulting materials. The authors mentioned that the variation in the concentration of P^5+^ and Ca^2+^ ions in the samples might be attributed to bone-like apatite on particles immersed in the culture medium. Nevertheless, *in vitro* bioactivity tests were not performed. It was determined that all of the evaluated compositions have antibacterial and bactericidal effects for *S. aureus* and *E. coli* and promote cell proliferation processes in human mesenchymal stem cells (hMSCs). According to the authors, these effects could be related to the combined action of Sr^2+^ and Zn^2+^ ions and the concentration of ZnO used in the samples. Particularly, the sample with X = 6, Y = 4 mol % presents a combined antibacterial and bactericidal effect along with enhanced cell proliferation. Nonetheless it seems that such effects are caused by the release and combined action of the ionic species from the materials.

Kaur et al. (2020)^75^ fabricated SrO-doped BGs from the composition 29.44SiO_2_-(45.55–X)­CaO-14.73MgO-10.28P_2_O_5_-XSrO (mol %) with X = 0, 1, 3, 5 mol % using the sol–gel method, followed by heat treatment at 700 °C for 5 h. The formation of Diopside (CaMgSi_2_O_6_) and Merwinite (Ca_3_Mg­(SiO_4_)_2_) was observed in all samples. However, with the increase in SrO, strontium phosphate (Sr_3_(PO_4_)_3_) was observed as an additional phase and an increase in the crystallinity of all samples. An increase in surface area (31–43 m^2^/g) and pore size (52–62 nm) was also observed as the SrO content increased. After *in vitro* tests, the precipitation of bone-like apatite was evident in all samples with SrO from day 1 of immersion in Tas’ SBF. However, the formation of this bioactive phase after 14 days of the test was greater in the samples with higher SrO content, which could be related to a greater surface area and larger pores, as reported by the authors. An increase in MG-63 osteoblast viability and cell proliferation was also reported with the increment of SrO content, attributed to the enhanced surface area and the beneficial effects of the released ions. However, no information related to the ionic release processes was presented.

On the other hand, Kermani et al. (2020)^76^ evaluated the effect of replacing CaO with SrO and CoO in BGs obtained from the composition: 41.2SiO_2_-5.06P_2_O_5_-(36.14–X–Y)­CaO-7.17Na_2_O-3.26MgO-7.17K_2_O-XSrO-YCoO, with (i) X = 0, Y = 0; (ii) X = 6, Y = 0; (iii) X = 0, Y = 1; (iv) X = 6, Y = 1 mol %. They fabricated the samples by sol–gel with a subsequent heat treatment at 580 °C for 4 h. Amorphous mesoporous particles with 2–50 nm pore sizes were obtained. It was determined that the addition of dopants promotes a decrease in particle sizes (684–308 nm) and in Z potential values (5–16 mV). After *in vitro* tests, the formation of bone-like apatite was observed in samples with SrO (X = 6, Y = 0 and X = 6, Y = 1 mol %) from 3 h of immersion in Kokubo SBF, while in the other samples, it occurred between 1–3 days of the test. Likewise, variations were observed in the concentrations of Ca^2+^, P^5+^, Si^4+^, Sr^2+^, and Co^2+^ ions released by the samples in SBF. As reported by the authors, all samples showed a greater release of Ca^2+^ ions during the test compared with the other ions that constitute the samples. Despite no structural data being reported by the authors that could explain such variations, the ion release test’s observations indicate that the presence of SrO in the compositions (as the only additive and added together with CoO) can induce bonding and structural changes in the glass network due to partial substitutions of Sr^2+^ and Co^2+^ in Ca^2+^ positions, thus affecting the solubility of the obtained BGs. Moreover, the addition of dopants did not significantly affect MG-63 osteoblast viability, likely because of the ions released from the samples.

Later, Swe et al. (2020)^77^ fabricated BGs from 53.85SiO_2_-(21.77–Y)­Na_2_O-1.72P_2_O_5_-(22.65–X)­CaO-XSrO-YAg_2_O, with (i) X = 0, Y = 0; (ii) X = 0, Y = 1; (iii) X = 3, Y = 0; (iv) X = 3, Y = 1 mol % by sol–gel, with subsequent heat treatment at 700 °C for 5 h. Dopant addition influenced the crystalline phases present in the samples. In the initial composition (X = Y = 0 mol %), an amorphous matrix was observed, and some crystalline contributions were associated with the formation of combeite (Na_2_Ca_2_Si_3_O_9_). This crystalline phase was observed in all of the fabricated samples. Amorphous contributions were also observed in the sample with X = 3 mol % SrO, along with disodium strontium phyllo-disilicate (Na_2_SrSi_2_O_6_) as an additional phase. While in the composition with X = 0, Y = 1 mol %, an increase in crystalline peaks and the formation of metallic silver (Ag) was evident as an additional phase. Finally, in the codoped sample, a greater crystalline contribution was observed, and the simultaneous formation of combeite, disodium strontium phyllo-disilicate, and metallic silver (Na_2_Ca_2_Si_3_O_9_, Na_2_SrSi_2_O_6_, and Ag, respectively). However, no crystallization mechanism of these phases was presented. In the *in vitro* tests, the formation of bone-like apatite was observed in all samples after 14 days of immersion in Hank’s solution (HBSS). However, the sample with 3 mol % SrO showed a greater formation from day 7 of the bioactivity test, which could be associated with different dissolution and precipitation processes of the samples. Now, in the ionic release assays in HBSS, a greater release of Sr^2+^ ions was evident in the codoped sample (X = 3, Y = 1 mol %), and a greater release of Ag^+^ ions in the sample with X = 0, Y = 1 mol % after 14 days of immersion in HBSS. However, no data was presented on the other ions in the compositions, thus limiting a deeper understanding of the dissolution phenomena of the samples. After the cellular assays, it was determined that the codoped sample promotes greater cell viability and proliferation in MC3T3-E1 osteoblasts and, in turn, inhibits the bacterial growth of *S. aureus* and *E. coli* compared to the other samples studied, which was associated with the effects induced by the joint action of Sr^2+^ and Ag^+^ ions as reported in the literature. However, these statements should be carefully interpreted due to the lack of dissolution data in this study.

Sharifianjazi et al. (2020)^78^ formulated BGs in the composition 60SiO_2_-4P_2_O_5_-(36–X–Y)­CaO-XSrO-YMgO mol % with (i) X = 0, Y = 5, 8, 10, and (ii) X = 5, 8, 10, Y = 0 mol %, presented in Figure 6a. These glasses were fabricated via the sol–gel method followed by heat treatment at 800 °C for 5 h. An increase in the Vickers microhardness and elastic modulus values was evident with the increase in the concentration of SrO and MgO in each group of samples. However, the elastic modulus values were higher for the MgO samples (90–112 GPa) than in the SrO samples (85–94 GPa). Meanwhile, Vickers microhardness values were higher for samples with SrO (460–485 HV) than those with MgO (430–450 HV). The authors attributed these variations to (i) the possible formation of Mg–O–Si and Sr–O–Si bonds and (ii) the fact that Sr^2+^ and Mg^2+^ ions can occupy interstitial sites within the glass network, improving the mechanical properties of the obtained BGs. After the bioactivity test using Kokubo SBF for 14 days, it was observed that the formation of bioactive phases in the samples was dependent on the type of oxide incorporated and the concentrations used. The formation of bone-like apatite was evident in all samples with MgO. However, in samples with SrO, a decrease in apatite precipitation was observed with increasing SrO, along with the formation of additional bioactive phases to the apatite depending on the SrO concentration. Particularly, in the sample with X = 8 mol % SrO, the presence of calcium orthosilicate (Ca_2_SiO_4_) was observed as an additional bioactive phase, and in the sample with X = 10 mol %, calcium orthosilicate (Ca_2_SiO_4_) and strontium silicate (SrSiO_3_) were formed as additional phases after immersion. Despite the authors not providing a possible explanation of the reduction of bone-like apatite formation together with the formation of the additional secondary bioactive phases (Ca_2_SiO_4_, SrSiO_3_), the presence of Ca_2_SiO_4_ under physiological conditions could indicate a possible competitive effect for Ca^2+^ between phosphate and silicate species in the SBF. Nonetheless, due to the lack of ion release data in this study, further studies should be carried out to provide detailed information about the possible mechanism associated with the formation of these bioactive phases in the SrO samples and the possible biological effects induced by these formed bioactive phases. Samples with X = 0 mol % SrO, Y = 8 mol % MgO and X = 5 mol % SrO, Y = 0 mol % MgO were selected for the cellular assays with G292 osteoblasts, because these samples presented greater formation of bone-like apatite as the only bioactive phase. From their results, they determined that the evaluated samples promote cell viability and proliferation processes. Nevertheless, the performance of the sample with 8 mol % MgO was higher than the one in the composition with 5 mol % SrO, which was associated with the effects induced by the ions present in the samples, although ion release analyses were not reported.

For their part, Huang et al. (2020)^79^ fabricated BG particles formulated from the composition 60SiO_2_-(36–X)­CaO-4P_2_O_5_-XSrO with X = 0, X = 6 mol % using sol–gel, with a subsequent heat treatment at 650 °C for 3 h as shown in Figure 6a. Particle size values of 401 and 534.6 nm and Z potential of 21.3 and 29.4 mV were reported for the compositions with X = 0 and X = 6 mol % SrO, respectively. In the ionic release assays in the DMEM cell medium, it was observed that the concentrations of Si^4+^, Ca^2+^, and P^5+^ ions were similar in both samples, while the concentration of Sr^2+^ ions was 5.518 mg/L in the sample with X = 6 mol % SrO. Cellular assays were then performed with RAW 264.7 macrophages, where it was shown that the composition with X = 6 mol % SrO promotes the inhibition of the osteoclast differentiation process due to the combined effect of Sr^2+^ and Si^4+^ ions in the disruption of several signal transduction processes: RANKL-activated p38 signaling and NF-kB signaling pathways, reported in the literature.

In turn, Moghanian et al. (2020)^80^ obtained BGs by sol–gel applying a subsequent heat treatment of 700 °C for 3 h from compositions formulated under the following conditions: 60SiO_2_-(31–X)­CaO-4P_2_O_5_-5ZrO_2_-XSrO, with X = 0, 3, 6, 9, and 12 mol %, as shown in Figure 6a. After the *in vitro* tests, the formation of bone-like apatite was evident in all samples after 14 days of immersion in Kokubo SBF. However, a decrease in the formation rate was observed for concentrations greater than X = 6 mol % SrO, where it is also proposed that Sr^2+^ ions could replace Ca^2+^ ions in the structure of the formed apatite. All of the BGs presented a similar trend in their dissolution processes according to the ion release graphs presented by the authors, with an increment in Si^4+^ and Ca^2+^ concentrations up to 3 days of testing and a subsequent reduction for further soaking times. For the P^5+^ species concentration, a trend to reduction with the increase of soaking time was evidenced, while Sr^2+^ and Zr^4+^ release presented an increment in their ionic release up to 7 days of testing, with no significant variations after 14 days of evaluation. However, the concentrations of Si^4+^, Ca^2+^ and Sr^2+^ were significantly higher at the end of the test, while the P^5+^ and Zr^4+^ concentrations were slightly higher (∼10 to 17 ppm and ∼0.4 to 0.6 ppm, respectively) after 14 days of soaking with increasing SrO content in the samples. Such findings could indicate a higher dissolution rate than the bioactivity rate due to structural modifications caused by SrO incorporation in the glass network. Moreover, since the concentrations of P^5+^ present in SBF (∼10–17 ppm after 14 days) are lower than those of Ca^2+^ (∼75–130 ppm), the nucleation and growing processes of the bone-like apatite formation could be affected, suggesting that the bioactive process is not always favored by a higher dissolution of the materials. In cellular assays with MC3T3-E1 osteoblasts, an increase in cell proliferation was observed as a function of the SrO content in the compositions up to X = 6 mol %, with a decrease for samples with higher SrO content. Likewise, it was shown that, although all samples with SrO promote an increase in ALP expression with respect to the initial composition, this effect is greater in the sample with X = 6 mol %. Finally, although all compositions with SrO presented a higher antibacterial performance against methicillin-resistant *S. aureus* (MRSA) compared to the initial composition (X = 0 mol %), the composition with X = 6 mol % promoted a superior antibacterial effect compared to the other samples evaluated. The authors attributed these effects to the increase in the ionic concentration of the samples, since the beneficial effects induced by the released ions are dependent on the concentrations in the physiological medium.

Later, Moghanian et al. (2021)^81^ fabricated BGs using sol–gel, followed by a heat treatment at 700 °C for 72 h, formulated from the composition: 60SiO_2_-(31–X)­CaO-4P_2_O_5_-5TiO_2_-XSrO, with X = 0, 1, 3, 6, 9, 12 mol %, shown in Figure 6a. The formation of bone-like apatite was observed after 7 days of immersion in Kokubo SBF. However, it was found that increasing SrO content delays the bone-like apatite formation process after 14 days of immersion in Kokubo SBF. From the dissolution graphs reported by the authors, it was evidenced that all samples exhibited similar tendencies in their ionic release profiles with increasing evaluation times: an increase in Si^4+^ concentrations was observed just after 1 day of soaking (∼30 ppm for all samples), with a tendency of decreasing concentrations with increment of soaking time. In the case of P^5+^ concentrations, a tendency to reduce with the increase of immersion time was evidenced, reaching values from 15–20 ppm after 14 days of evaluation. Moreover, despite all samples exhibiting similar concentration values of Ca^2+^ after 1 day of testing (∼300 ppm), a rapid decrease was observed from 3 to 7 days of soaking, with no significant variations after 14 days of immersion. The Sr^2+^ and Ti^4+^ ionic release exhibited a similar trend: an increment up to 3 days of soaking, with no significant variations for higher evaluations times. Nonetheless, despite the similar tendencies in their release profiles, a significant increase in ionic concentrations of Si^4+^, Ca^2+^ and Sr^2+^ alongside a decrease in Ti^4+^ concentration with the increment of SrO content was evidenced after 14 days of immersion in Kokubo SBF; while the P^5+^ species concentration only exhibited slight increments for higher SrO concentrations during the test. These observations suggest a higher dissolution rate than the bone-like apatite rate in the materials caused by SrO incorporation. Despite the increased concentration of Ca^2+^ ions in the physiological media, the bioactive behavior of the materials is dependent on the P^5+^ concentrations available in the SBF (15–20 ppm after 14 days) to promote the nucleation and growing processes of the bone-like apatite layer. Such findings are indicative that a higher dissolution rate does not promote an accelerated formation of bone-like apatite. Cellular assays show that SrO addition affects the MC3T3-E1 osteoblasts proliferation differently: concentrations up to X = 6 mol % promote increased proliferation, while higher SrO concentrations induce cytotoxic effects. Additionally, antibacterial performance against MRSA improved with increasing the SrO content. These effects are likely related to the increased release of ions in samples with higher SrO content, potentially exceeding beneficial concentration thresholds reported in the literature.

Özarslan and Yücel (2022)^82^ evaluated the effect of simultaneous addition of SrO and CuO in BGs formulated from the composition: 51.21SiO_2_-(26.33–X)­CaO-(19.86–Y)­Na_2_O-2.60P_2_O_5_-XSrO-YCuO mol %, named C1: X = Y = 0; C2: X = 2.42, Y = 0; C3: X = 2.42, Y = 0.08; C4: X = 2.42, Y = 0.16; and C5: X = 2.42, Y = 0.24 mol %, which were obtained by the melt-quenching method. The authors reported density values of 2.670 g/cm^3^ for the C1 composition and values of 2.710, 2.715, 2.724, and 2.727 g/cm^3^ for samples C2–C5, respectively. An increase in Vickers hardness values was also determined: 350 HV for the C1 BG, and 410, 415, 426, and 433 HV for the C2–C5 samples, respectively. Values of*T*_g_ = 545 °C, *T*_c_ = 849 °C, and working temperature *T*_w_ = 225 °C were reported for the C1 composition, and variations in the values of these parameters with the simultaneous addition of SrO and CuO, with *T*_g_ = 543, 546, 546, and 547 °C; *T*_c_ = 883, 886, 879, and 879 °C; *T*_w_ = 239, 245, 247, 245 °C for the C2–C5 compositions, respectively. These values are higher compared to those reported for traditional compositions, such as 45S5 and S53P4, as shown in Figure 7. Compared to the base composition, the addition of SrO (sample C2) promoted an increment in the release of Si^4+^, Ca^2+^, Na^+^, P^5+^, and Sr^2+^ species in Tris-HCl solution at 37 °C, suggesting changes in the glass network affecting the dissolution behavior of this sample. However, the CuO content led to a decrease in the concentration of Si^4+^, Ca^2+^, Na^+^, P^5+^, and Sr^2+^ ions in Tris-HCl solution at 37 °C, accompanied by an increase in Cu^2+^ ion concentration compared to the initial sample. This suggests that, despite the lower content, the addition of CuO reduces the dissolution rate of the compositions, due to possible structural modifications caused by Cu^2+^ presence in substitutional and interstitial positions in the glass network. The authors related the variations in the thermal and mechanical parameters and the changes in the dissolution kinetics with possible changes in the NBOs of the glassy network due to the incorporation of SrO and CuO. Initially, the incorporation of SrO to the base composition can induce structural and bonding modifications by causing a slight reduction in the network connectivity, but increasing mechanical properties, favoring thermal parameters such as *T*_c_ and *T*_w_, and promoting a high dissolution rate; while the dual presence of SrO and CuO can cause another structural effect: increasing in network connectivity due to Sr^2+^ and Cu^2+^ presence in substitutional and/or interstitial positions, thus incrementing mechanical properties, and favoring thermal parameters but delaying the ion release behavior of the samples. This is a useful contribution providing insights not only on the specific role of SrO addition but also on the multiple effects induced by the dual action of SrO and lower concentration of CuO.

Anand et al. (2022)^83^ obtained amorphous mesoporous particles of BGs codoped with CuO and SrO, which were formulated from the composition 80SiO_2_-(15–X–Y)­CaO-5P_2_O_5_-XCuO-YSrO with X = Y = 0, 0.5, 1, and 2 mol % as shown in Figure 6. b). The compositions were manufactured using the EISA method, which applied a subsequent heat treatment at 700 °C for 5 h. In the ion release test, differences in the dissolution processes of the resulting materials were evidenced in the graphs presented. Initially, Cu^2+^ and Sr^2+^ species exhibited similar trends, with an increase in ion release up to 7 days of soaking and no significant differences in concentrations after 14 days of testing. Despite the similar tendency, higher concentration values were observed in samples with higher CuO and SrO content. In the case of Si^4+^ species, an increment in concentrations was observed up to 1 day of evaluation with a posterior reduction for superior immersion times, with slight variations in the concentration values after 14 days (∼62 mg/L); while the P^5+^ concentrations presented a tendency to reduction up to 7 days of soaking, with no significant variations after 14 days of the test reaching concentrations between ∼1–4 mg/L at the end of the test. In contrast, the Ca^2+^ release profiles were different among the samples. The base composition (X = Y = 0 mol %) exhibited a slight increment up to 1 day of soaking, with no significant variations for further evaluation times, with values ∼45 mg/L, while the samples X = Y = 0.5 mol % and X = Y = 2 mol % presented similar tendencies, incrementing the Ca^2+^ concentration up to 7 days of testing, with a subsequent reduction for further testing times, reaching concentrations ∼65 and ∼35 mg/L, respectively. However, the X = Y = 1 mol % sample did not exhibit significant variations in the released concentrations (∼57 mg/L) through the test. Such variations in the release profiles of the samples could be related to the dual effects of SrO and CuO in the structure and bonding of the glass network. However, due to the lower concentration introduced in the samples, Sr^2+^ and Cu^2+^ ions could partially substitute Ca^2+^ positions inside the glass matrix and also can occupy interstitial positions, thus affecting the dissolution process of the samples. But, since no structural information on the network connectivity was reported, such interpretations should be carefully addressed by the readers. In the bioactivity assays in Kokubo SBF, it was evident that the presence of codopants delays the formation of bone-like apatite since in the compositions with X = Y = 0 mol % and X = Y = 0.5 mol % the precipitation of this bioactive phase was observed from 8 h of immersion, while for the samples with higher SrO and CuO contents, the formation of apatite was evident between 1–7 days of immersion, which could be related to the differences in ionic release reported by the authors, especially with the Ca^2+^ concentrations released by the samples, since the bone-like apatite formation is dependent on Ca^2+^ and phosphate species concentrations. It was evident from the cellular assays that with the increase in the codopant’s concentrations, a decrease in cell viability induced by extracts of the samples in MG-63 osteoblasts is promoted. However, the sample with X = Y = 0.5 mol % showed a better cellular performance compared to the other compositions. An increase in bacterial growth inhibition was also observed in *E. coli* and *S. aureus* as a function of the codopant concentration. These effects could be associated with the high concentrations of ions released by the samples, which could induce toxic responses in the cells, as reported in the literature.

On the other hand, Ningsih et al. (2022)^84^ fabricated BGs formulated from the composition 60SiO_2_-(35–X)­CaO-5P_2_O_5_-XSrO, with X = 0, 1, 3, 5 mol %, by the spray-drying method, with a subsequent heat treatment at 600 °C for 1 h, shown in Figure 6b. As reported, amorphous microspheres were obtained with variations in morphologies associated with SrO content. Although well-defined spherical particles were evident in all samples, for X = 0 and 1 mol %, particles with concave morphologies were observed. In contrast, wrinkled particles were present for X = 3 and 5 mol %, which could suggest a dependence on the composition and the resulting morphology of the particles. Following *in vitro* tests, bone-like apatite formation was observed in all samples after 7 days of immersion in Kokubo SBF. Also, an increase in cell viability using MC3T3 osteoblast was determined in the SrO-doped samples compared to the initial composition (X = 0 mol % SrO), which presented viability <70% and is cytotoxic according to ISO 10993–5. The authors attributed these responses to the effects induced by the ions released by the compositions but did not present ion release results from the samples.

Oliveira et al. (2022)^85^ prepared a BG composition by sol–gel, applying a postheat treatment at 550 °C for 2 h, and evaluated the effect of the phosphorus precursor (PA-phosphoric acid vs TEP-triethylphosphate) and the NH_4_OH concentration (1 vs 2 M) on the physical and chemical properties of the manufactured BGs. From the results obtained, they selected the precursor’s PA and 1 M NH_4_OH to prepare BGs formulated from the composition: 60SiO_2_-(36–X)­CaO-4P_2_O_5_-XSrO with X = 0 and 3 mol % using the mentioned methodology (sol–gel and heat treatment at 550 °C for 2 h), presented in Figure 6. a). The results suggest that the presence of SrO does not generate significant changes in the glass network of the sample with X = 3 mol %, based on the changes in Raman bands intensity. However, no quantitative data associated with the network structure was presented by the authors. Following *in vitro* evaluations, the precipitation of bone-like apatite and calcite (CaCO_3_) was observed as bioactive phases from day 1 of the test for both samples, with a higher presence of calcite in the compositions after 14 days of immersion in Kokubo SBF. Also, it was observed that the addition of X = 3 mol % SrO promotes an increase in the precipitation of bone-like apatite compared to the initial composition (X = 0 mol % SrO). These effects could be associated with structural and bonding modifications caused by Sr^2+^ incorporation in substitutional or interstitial positions in the glass network, possibly affecting the dissolution behavior of the materials. However, these statements should be carefully interpreted due to the lack of ion release information and quantitative structural data in this study. In indirect cellular assays with SaOS-2 osteosarcoma, where concentrations of 100, 50, 25, 12.5, and 6.25 mg/mL were used, the initial composition was observed to promote cell viability up to 25 mg/mL. In comparison, the sample with X = 3 mol % SrO presents a higher cell viability up to 50 mg/mL, which could be associated with the effect of the ions released by the samples. However, no dissolution data on the compositions was reported, as already mentioned.

Özarslan and Yücel (2023)^86^ evaluated the dissolution behavior and bioactive response of the compositions previously developed in^82^ by immersing them in Kokubo’s SBF for up to 28 days. Initially, the C2 composition (2.42 mol % SrO) presented a higher concentration of Si^4+^, Na^+^, and Sr^2+^ together with a rapid release and reduction of Ca^2+^ and P^5+^ species released during the soaking period in SBF compared to the based composition and the other evaluated samples, suggesting an accelerated dissolution process accompanied by a high bioactive response due to the SrO addition. However, the increase of the CuO content in the samples led to a decrease in the concentrations of released Si^4+^, Na^+^, and Sr^2+^ ions, together with a slow release of Ca^2+^ and P^5+^ species, while increasing the concentration of released Cu^2+^ ions over the 28-day period. This behavior was associated with changes in the number of nonbridging oxygens (NBOs) within the glass network, which in turn influenced the dissolution process. Despite SrO addition improving the dissolution of the composition, it seems that dual SrO and CuO presence can cause a delay in the ion release behavior. Although all samples demonstrated the formation of bone-like apatite after the bioactivity tests, the C2 sample (2.42 mol % SrO) promoted an accelerated precipitation compared with the base composition and the other evaluated samples. These results highlight that SrO incorporation specifically promoted an accelerated dissolution process with a rate that seems to match the rapid formation rate of bone-like apatite due to the interactions of the Ca^2+^ and phosphate species in the SBF. However, the presence of CuO appeared to reduce the precipitation rate of this bioactive phase, despite the presence of SrO in the samples. These observations provide meaningful insights into the single effect of SrO and the dual SrO and CuO presence in the formulated materials.

Subsequently, Simon et al. (2024)^87^ synthesized BGs using the sol–gel method, followed by thermal treatment at 550 °C for 1 h. These glasses were based on the composition 60SiO_2_-5P_2_O_5_-(35–X)­CaO-XSrO, with X = 0, 2, 5, and 10 mol % as presented in Figure 6b. Their findings revealed that the incorporation of SrO did not disrupt the amorphous structure of the glass network. After *in vitro* evaluation, bone-like apatite precipitation was observed in the samples with X = 5 and 10 mol % SrO after 7 days of immersion in Kokubo SBF. In contrast, no formation of this phase was observed in samples with X = 0 and 2 mol % SrO during the test period. Antibacterial assays demonstrated enhanced activity against *Streptococcus* sp. and *E. coli* with increasing SrO content. These effects were attributed to changes in the dissolution behavior due to structural modifications of the glass network. However, the authors did not report the ionic release data for these compositions.

On the other hand, when the substitution of CaO by SrO exceeds 50%, distinct effects on the BG structure may arise, and these compositions are summarized in Table 3. Lee et al. (2019)^88^ developed a BG system with the composition 46.1SiO_2_-24.4Na_2_O-(26.9–X–Y)­CaO-2.6P_2_O_5_-XSrO-YMgO (mol %), with the following conditions: X = Y = 0 mol %; X = 26.9, Y = 0 mol %; and X = 0, Y = 26.9 mol %. The samples were fabricated via melt-quenching. Their results indicated structural changes in the glass network associated with the distribution of the *Q*_Si_^*n*^ units. Specifically, the sample with Y = 26.9 mol % MgO exhibited approximately 72.5% *Q*_Si_^3^, 14.5% *Q*_Si_^2^, 10.6% *Q*_Si_^1^, and 2.4% *Q*_Si_^0^, indicating greater continuity in the silicate network compared to the initial composition (X = Y = 0 mol %) and the sample with X = 26.9 mol % SrO. The latter showed reduced contributions from *Q*_Si_^3^ (47.6% and 48.0%, respectively) and *Q*_Si_^1^ (1.4% and 7.3%, respectively), with increased fractions of *Q*_Si_^2^ (42.8% and 36.1%, respectively) and *Q*_Si_^0^ (8.3% and 8.5%, respectively). These results suggest that MgO acts as a network former, enhancing network connectivity in the sample with X = 0, Y = 26.9 mol %. In this study, the BG compositions were also incorporated into poly­(lactic acid) (PLLA) polymeric matrices in the form of electrospun fibers.

In contrast, Al-Khafaji et al. (2019)^89^ developed a series of bioactive glass (BG) compositions based on the system 35.5SiO_2_-7.5Na_2_O-(45–X)­CaO-6P_2_O_5_-(6–Y)­CaF_2_-XSrO-YSrF_2_ (mol %). Three series were established as follows: Series I with X = 0; Y = 0, 3, and 6 mol %, Series II with X = 22.5; Y = 0, 3, and 6 mol %, and Series III with X = 45; Y = 0, 3, and 6 mol %. The compositions were fabricated via melt-quenching. The results revealed a crystallization trend that was dependent on the SrO content. Series I samples exhibited an amorphous structure, whereas Series II samples showed crystalline contributions from calcium and strontium fluorapatites: (Ca_5_(PO_4_)_3_F) and (Sr_5_(PO_4_)_3_F), respectively, embedded in a glassy matrix. Series III samples presented a crystalline matrix composed mainly of strontium fluorapatite (Sr_5_(PO_4_)_3_F) with some amorphous regions. However, the authors did not provide mechanisms for the formation of these phases or quantitative data regarding their content. In dissolution tests conducted in Tris-buffered saline (TBS), a rapid release of P^5+^ ions were observed after 3 h of immersion, with higher concentrations recorded for Series II samples. After this initial period, a sharp decrease in ion concentrations was noted in Series I, while Series II and III samples exhibited a gradual decline over longer immersion times. Regarding F^–^ ions, the concentration trend followed the order Series II > Series I > Series III, attributed to the formation of poorly soluble phases such as strontium and calcium fluorapatites: (Sr_5_(PO_4_)_3_F and Ca_5_(PO_4_)_3_F), respectively. Nevertheless, no data was provided for other ions present in the compositions. The ability to form bioactive phases was also evaluated after 168 h in TBS for the selected samples: X = Y = 0 mol % and X = 0 mol %, Y = 6 mol % from Series I; X = 22.5 mol %, Y = 3 mol % from Series II; and X = 45 mol %, Y = 3 mol % from Series III. Variations in the concentrations of Ca^2+^, F^–^, P^5+^, and Sr^2+^ ions over time were reported, indicating the formation of bioactive phases. In particular, Series I samples showed bone-like apatite precipitation between 3 and 6 h of immersion, correlating with a decrease in P^5+^ and Ca^2+^ concentrations. In contrast, Series II and III samples exhibited an increased intensity of crystalline phase peaks, while their ionic concentration changes were less pronounced. These differences in dissolution behavior were attributed to the presence of poorly soluble crystalline phases in Series II and III, as opposed to the more soluble amorphous nature of the Series I samples.

Terzopoulou et al. (2019)^90^ fabricated bioactive glass (BG) compositions of 80SiO_2_-(10–X)­CaO-XSrO-10P_2_O_5_, with X = 0 and 10 mol %, via a hydrothermal route followed by thermal treatment at 600 °C for 5 h. The resulting samples exhibited a mesoporous character, with pore sizes of 3.3 and 3.5 nm for X = 0 and X = 10 mol %, respectively. While the initial composition displayed an amorphous structure, the sample with X = 10 mol % showed the presence of strontium silicate (Sr_2_SiO_4_) and strontium carbonate (SrCO_3_) embedded in a glassy matrix. After evaluating bioactivity in Kokubo’s SBF for 14 days, the formation of bone-like apatite and strontium apatite was observed in the X = 0 and X = 10 mol % compositions, respectively, as early as the first day of testing. However, the sample with X = 10 mol % exhibited superior bioactive performance. Additionally, both samples were found to promote the proliferation of Wharton’s jelly derived mesenchymal stem cells (WJ-MSCs). Such structural effects and the bioactive performance of the samples could be related to structural changes in the glass network caused by the total substitution of SrO for CaO. However, the knowledge about these possible effects was limited due to the lack of structural information (related to *Q*_Si_^*n*^ units) and dissolution data in the study.

Fei et al. (2021)^91^ fabricated mesoporous spherical BG particles based on the composition 80SiO_2_-(15–X)­CaO-5P_2_O_5_-XSrO, with X = 0, 1, 5, and 10 mol %, using the spray pyrolysis technique, shown in Figure 6b. In the sample with X = 10 mol %, a predominantly amorphous structure was observed, along with crystalline peaks corresponding to strontium silicate (Sr_2_SiO_4_) and strontium pyrophosphate (Sr_2_P_2_O_7_). The other compositions remained amorphous. However, the authors did not provide quantitative data or mechanisms related to the formation of these crystalline phases. After 7 days of immersion in Kokubo’s SBF, bone-like apatite formation was observed in the reference composition (X = 0 mol %), while samples containing SrO exhibited both bone-like apatite and strontium-substituted apatite as bioactive phases. Ionic release tests conducted in phosphate-buffered saline (PBS) revealed variations in the Sr^2+^ ion concentrations among the compositions. The sample with X = 5 mol % exhibited the highest release, followed by those with X = 1 mol % and X = 10 mol %, with the latter releasing the lowest Sr^2+^ concentration. This behavior was attributed to the presence of crystalline phases with low solubility in the sample with X = 10 mol %, which likely limited Sr^2+^ ion release. However, the authors did not report ionic concentration data for the other constituents of the compositions, limiting the scope of this study. Finally, in biological assays using human bone marrow-derived mesenchymal stem cells (hBMSCs), an increase in cellular activity was observed for samples containing up to X = 5 mol %, followed by a decrease in activity for the sample with X = 10 mol %. This trend was associated with the varying concentrations of Sr^2+^ ions released from the different compositions. However, the *in vitro* performance of the materials should be related to the multiple effects caused by the ionic species released from the BGs.

Mosqueira et al. (2021)^92^ synthesized spherical BG particles based on the composition 80SiO_2_-(16–X)­CaO-4P_2_O_5_-XSrO, with X = 0, 5, 10, and 16 mol %, using the sol–gel method followed by thermal treatment at 650 °C for 3 h, as shown in Figure 6a. Their results showed that SrO substitution led to an increase in both particle size and pore size compared with the base composition. Additionally, all samples exhibited crystalline phases embedded within an amorphous matrix, with the crystalline phases varying according to SrO content: (i) calcite (CaCO_3_) was identified in samples with X = 0 and 5 mol %, possibly formed via reaction with atmospheric CO_2_ during synthesis; (ii) strontium silicate (Sr_2_SiO_4_) and strontium carbonate (SrCO_3_) were observed in samples with X = 10 and 16 mol %. However, no quantitative analysis or mechanistic explanation for the formation of these crystalline phases was provided. Regarding ionic release studies conducted in Kokubo’s SBF, similar trends were observed in the graphs of the samples: an increment in Si^4+^ concentrations up to 1 day of soaking, with no significant changes for higher evaluation times. Moreover, no significant variations in Si^4+^ ion concentration with an increase in SrO content were detected. In the case of Ca^2+^ species, the sample with X = 10 mol % presented a rapid concentration increase up to 1 day of testing, while the other samples exhibited a similar trend up to 8 h of immersion. After that, all samples showed a tendency to reduce the ionic concentration until the end of the test. However, the Ca^2+^ concentration values exhibited a decrease with increasing SrO, as expected. The P^5+^ concentrations presented different behavior depending on the SrO content: the X = 0 and 5 mol % samples showed a rapid reduction tendency from the beginning to the end of the test, reaching similar values from 7 to 14 days of soaking (∼2.5 ppm). However, the X = 10 and 16 mol % BG exhibited no significant variations in concentration up to 1 day of immersion, with a subsequent reduction until the end of the test, with values ∼3 and 10 ppm, respectively, after 14 days of testing. In addition, an increase in P^5+^ concentrations with the increase of SrO content was evidenced from 3 days of soaking until the end of the test. In the case of Sr^2+^ species, a fast release up to 1 day of immersion was observed in all samples, with a slight reduction for further evaluation times in samples X = 5 and 10 mol %, together with an increase after 7 days of soaking, with no significant variations up to 14 days of testing for the X = 16 mol %. Moreover, an increment in Sr^2+^ concentrations with the increase of SrO content in the samples was evidenced, as expected. Bioactivity tests in SBF demonstrated that the formation of bioactive phases was dependent on the SrO content. Bone-like apatite formation was observed in samples with X = 0 and 5 mol %, whereas samples with higher SrO content (X = 10 and 16 mol %) showed distinct behaviors: (i) a marked reduction in peaks associated with strontium silicate (Sr_2_SiO_4_), indicating potential dissolution of this phase; and (ii) precipitation of strontium apatite and strontium carbonate (SrCO_3_) as bioactive phases after 14 days of immersion. Those observations suggest a complex dissolution process accompanied by the precipitation of different bioactive phases, which was not addressed in this study. While the X = 0 and X = 5 mol % samples exhibited the typical formation of bone-like apatite, the SrO for CaO substitution above 50% seems to affect the *in vitro* performance of the X = 10 and 16 mol % materials. Considering the different dissolution rates between the glassy matrix and the strontium silicate (Sr_2_SiO_4_) phase, the ionic availability of Si^4+^ species in the SBF reflects the overall dissolution of the biphasic material (amorphous matrix with crystalline contributions) rather than the overall glass matrix dissolution. Finally, in cellular assays using bone marrow-derived mesenchymal stem cells (BMSCs) from Wistar rats, all extracts from the evaluated compositions exhibited osteogenic potential. Notably, extracts from the composition containing 10 mol % SrO showed superior osteogenic effects compared to the other samples, which may be attributed to the influence of the released ionic species. Thus, the results reported by the authors could suggest that SrO incorporation alters not only the structural features of the materials but also the *in vitro* performance of the BG compositions.

The evidence discussed in the studies summarized in Table 3, suggests that SrO by CaO substitution above 50% could affect the amorphous nature of BGs due to the formation of crystalline contributions embedded in a glassy matrix. Moreover, the *in vitro* performance of the materials can be affected depending on the reactivity of such crystalline formations since some of the phases could present an inert behavior, while others could have a dissolution rate lower than the dissolution rate of the glassy network. Interestingly, the reduction of CaO content in BGs does not seem to inhibit the bioactive behavior when samples are soaked in physiological media, since the precipitation of strontium apatite and/or other Sr-containing phases under physiological conditions has been reported. However, further *in vitro* and *in vivo* approaches should be carried out to provide evidence of possible effects caused by the presence of such bioactive phases.

While numerous studies have focused on the incorporation of SrO into BG systems to enhance thermal, mechanical, and biological responses, a few have examined the opposite approach—replacing SrO with other oxides. Although SrO is not the variable of interest in such cases, these studies offer useful comparative insights into SrO’s contribution by revealing how its absence or substitution alters glass behavior. These comparative studies are summarized in Table 4. In this context, Goudarzi et al. (2019)^93^ examined the effect of substituting SrO with ZnO on the composition, structure, and performance of BGs formulated from the system 60SiO_2_-4P_2_O_5_-31CaO-(5–X)­SrO-XZnO mol %, with X = 0, 1, 2, 3, 4, and 5 mol %, included in Figure 6a. The materials were synthesized by using the sol–gel method followed by thermal treatment at 580 °C for 5 h. Their findings indicated that SrO reduction did not alter the amorphous character of the resulting materials. *In vitro* bioactivity evaluations revealed a progressive reduction in bone-like apatite formation with decreasing SrO and increasing ZnO content after 21 days of immersion in Kokubo SBF. Notably, the sample with X = 1 mol % (higher SrO content) exhibited the earliest formation of this bioactive phase, detected after just 3 days of immersion. These results suggest that SrO reduction by ZnO incorporation causes adverse effects on the bioactive performance of the materials. Ionic release tests in SBF were conducted for samples with ZnO contents ranging from 1 to 4 mol %. For Sr^2+^ and P^5+^ ions, a decrease in release concentrations was observed as ZnO content increased, consistent with the reduction of SrO. Conversely, the concentration of Zn^2+^ ions increased proportionally with ZnO incorporation. The behavior of Si^4+^ and Ca^2+^ ion release showed more complex trends. In the case of Si^4+^, higher concentrations were initially observed in samples with X = 1 and 2 mol %, but from day 3 onward, samples with higher ZnO (lower SrO) contents exhibited greater ion release, a trend that persisted until day 21. For Ca^2+^, lower concentrations were recorded on day 1 as the ZnO content increased or SrO decreased. On day 3, a significant drop in Ca^2+^ concentration was observed for the X = 1 mol % sample, while other compositions showed reduced concentrations by day 7. From that point forward, higher Ca^2+^ release was observed in samples with a higher ZnO content (lower SrO). These fluctuations in Ca^2+^ and P^5+^ concentrations were attributed to bone-like apatite formation and precipitation processes. These observations indicate a complex dissolution process in the samples probably related to changes in the glass matrix due to the ZnO incorporation by SrO. However, no structural data related to the network connectivity was presented in this study. Antibacterial assays demonstrated that all compositions containing 1–5 mol % ZnO inhibited the growth of *S. aureus*, with the X = 2 mol % composition exhibiting the strongest antibacterial effect. This was likely due to the synergistic influence of the released ions. Additionally, cytotoxicity assays with G292 osteoblasts indicated a decrease in cell viability with increasing ZnO content, although the X = 1 mol % sample displayed the most favorable performance. This behavior was similarly attributed to the combined action of the different ions released by the different compositions. The overall *in vitro* performance of the compositions reported in this study suggest that substituting ZnO for SrO can cause several adverse effects in the materials, providing evidence of the importance of the SrO presence in the performance of BGs.

**Table 4:** BG Formulations Where SrO Is Substituted by Other Oxides

glass composition (mol %)						*in vitro* bioactivity			cell assays		bacterial assays		
SiO_2_	CaO	P_2_O_5_	SrO	other oxides	structural features	solution	immersion times	bioactive phases	cell line	significant results	bacterial strain	significant results	ref
60	31	4	5		amorphous	Kokubo’s SBF	3, 7, 14, and 21 days	bone-like apatite formation	osteoblast-like G292 cells	cell viability	*S. aureus*	antibacterial activity	^93^
60	31	4	4	1 (ZnO)	amorphous			decrease in apatite formation with ZnO increasing		decrease in cell viability with ZnO increasing		antibacterial activity	
60	31	4	3	2 (ZnO)	amorphous							antibacterial activity	
60	31	4	2	3 (ZnO)	amorphous							antibacterial activity	
60	31	4	1	4 (ZnO)	amorphous							antibacterial activity	
60	31	4	0	5 (ZnO)	amorphous							antibacterial activity	
42	34	6	18		amorphous	Kokubo’s SBF	14 and 28 days	decrease in apatite formation with Al_2_O_3_ increasing	human osteosarcoma cells U2-OS	cell proliferation and viability	not reported		^94^
42	34	6	17.5	0.5 (Al_2_O_3_)	amorphous					cell proliferation and viability			
42	34	6	17	1 (Al_2_O_3_)	amorphous					cell proliferation and viability			
42	34	6	16.5	1.5 (Al_2_O_3_)	amorphous					cell proliferation and viability			
42	34	6	15.5	2.5 (Al_2_O_3_)	amorphous					cell proliferation and viability			
45	18.5	6	12	18.5 (Na_2_O)	crystalline phases	not reported			not reported		*S. aureus*	antibacterial activity	^95^
45	18.5	6	8	18.5 (Na_2_O), 4 (CuO)	crystalline phases							superior antibacterial activity	
45	18.5	6	4	18.5 (Na_2_O), 8 (CuO)	crystalline phases							antibacterial activity	
45	18.5	6	0	18.5 (Na_2_O), 12 (CuO)	crystalline phases							antibacterial activity	

Tripathi et al. (2019)^94^ investigated the effect of incorporated Al_2_O_3_ for SrO in BGs with the composition 42SiO_2_-34CaO-6P_2_O_5_-(18-X)­SrO-XAl_2_O_3_ mol %, where X = 0, 0.5, 1, 1.5, and 2.5 mol %, synthesized using the melt-quenching technique. All compositions maintained an amorphous structure. An increase in density, compressive strength, and elastic modulus were observed with increasing Al_2_O_3_ and corresponding reduction in SrO content. These enhancements were attributed to the incorporation of Al^3+^ ions into interstitial positions within the glass network, consistent with the previous literature. *In vitro* bioactivity assessments revealed that the formation of bone-like apatite increased with Al_2_O_3_ content up to X = 1.5 mol %, whereas a reduction in this phase was observed for the X = 2.5 mol % composition after 28 days of immersion in Kokubo SBF., suggesting a reduction in the bioactive behavior due to lower SrO content. Furthermore, all compositions promoted cell viability in human osteosarcoma cells (U2-OS) and peripheral blood mononuclear cells (PBMCs). Hemolysis tests confirmed that none of the samples induced hemolytic activity in red blood cells (RBCs). The authors suggested that the observed biological responses were likely influenced by modifications in the glass network structure due to Al_2_O_3_ incorporation together with SrO content reduction and the bioactivity associated with the elements present in the glass formulations. However, no data on the ionic release profiles of the evaluated samples were provided. The findings reported by the authors suggest that the reduction of SrO by Al_2_O_3_ incorporation should be carefully addressed, since the mol % of Al_2_O_3_ selected could affect the bioactivity performance of the resulting materials due to the structural modifications caused in the glassy network affecting the performance of the materials.

Perry and Wren (2024)^95^ developed the BG where CuO replaces SrO in which compositions 45SiO_2_-6P_2_O_5_-18.5CaO-18.5Na_2_O-(12–X)­SrO-XCuO mol %, where X = 0, 4, 8, and 12 mol %, were synthesized via sol–gel with a thermal treatment at 600 °C for 2 h. Their findings indicated that the reduction of SrO by addition of CuO induced (i) a progressive decrease in glass transition temperatures (*T*_g_), 561 °C, 555 °C, 547 °C, and 543 °C, and crystallization temperatures (*T*_c_), 917 °C, 823 °C, 820 °C, and 791 °C, for samples with X = 0, 4, 8, and 12 mol %, respectively; and (ii) variations in melting temperatures (*T*_m_), 1132 °C, 915 °C, 945 °C, and 1137 °C, corresponding to the same CuO concentrations. These thermal behaviors were associated with changes in the number of nonbridging oxygens (NBOs) in the glass network due to the incorporation of Cu^2+^ ions, associated with the variations in intensity of the Raman bands related to *Q*_Si_^*n*^ units. Nonetheless, the understanding of the effect of SrO substitution on the glass network by CuO was limited, since no quantitative analysis of Raman spectra was provided in this study. All samples exhibited an amorphous matrix with crystalline contributions, with the crystalline phases varying according to the CuO and SrO content. Specifically, calcium strontium phosphate and calcium phosphate silicate were observed in the X = 0 mol % sample; calcium strontium phosphate, strontium calcium copper phosphate, calcium phosphate silicate, and CuO in the X = 4 mol % sample; strontium calcium copper phosphate and CuO in the X = 8 mol % sample; and calcium copper phosphate and CuO in the X = 12 mol % sample. The authors suggested that the formation of these crystalline phases could be related to the thermal treatment temperature exceeding *T*_g_, rather than to a possible effect caused by the CuO for SrO substitution, although no crystallization mechanisms based on CuO or SrO content were discussed. In dissolution tests, using deionized water and conducted over 1000 h, the authors observed that increasing CuO by decreasing SrO content led to (i) a decrease in Sr^2+^ ion release, as expected, and an increase in P^5+^ ion concentration; (ii) variable behavior in Si^4+^, Ca^2+^, and Na^+^ ion release; and (iii) no detectable Cu^2+^ ion release, attributed to the presence of crystalline CuO in samples with X = 4, 8, and 12 mol %. Notably, the compositions with X = 0 and 4 mol % CuO released higher concentrations of Si^4+^, Ca^2+^, and Sr^2+^ ions and exhibited higher pH values compared to the other compositions. Finally, antibacterial assays revealed that all compositions inhibited the growth of *S. aureus*, with the sample containing X = 4 mol % CuO showing superior antibacterial activity. This was attributed to the elevated pH and the synergistic effects of the ions released during dissolution. However, the coexistence of amorphous and crystalline contributions in the materials significantly impacted the overall performance of these samples.

The articles summarized in Table 4 provide indirect but valuable evidence of SrO’s role by highlighting how its removal or substitution modifies glass properties. Depending on the type of oxide and concentration used to substitute SrO, different effects were evidenced: (i) negatively impacting the *in vitro* performance of the materials when using ZnO; (ii) improving mechanical properties of the glasses but delaying the bone-like apatite formation for Al_2_O_3_ addition. Thus, offering insights into future research toward this field.

Although partial and total substitutions of CaO with SrO are the most frequently reported in the literature, several studies have explored the use of SrO as a replacement for other oxides in BG compositions, as summarized in Table 5. In this context, Jiménez-Holguín et al. (2020)^96^ investigated the incorporation of SrO as a substitute for SiO_2_ in BGs with the composition (85–X)­SiO_2_-10CaO-5P_2_O_5_-XSrO mol %, where X = 0, 2.5, and 5 mol %, presented in Figure 6b. The materials were synthesized via the EISA technique, followed by a thermal treatment at 700 °C for 6 h. Their findings revealed a reduction in particle size, pore volume, and surface area with increasing SrO content. Additionally, structural changes in *Q*_Si_^*n*^ units were reported based on single-pulse magic angle spinning nuclear magnetic resonance (SP MAS NMR) spectra: a marked decrease in *Q*_Si_^4^ units (89.2%, 76.8%, and 48.7%) and *Q*_Si_^2^ units (6.0%, 1.0%, and 1.0%), accompanied by an increase in *Q*_Si_^3^ units (4.6%, 22.1%, and 50.2%) for compositions with X = 0, 2.5, and 5 mol %, respectively. Regarding phosphate units, there was an increase in *Q*_P_^0^ contributions (93.4%, 95.6%, and 96.5%) and a corresponding decrease in *Q*_P_^1^ units (6.5%, 4.3%, and 3.4%) with an increasing SrO content. These results suggest that SrO acts as a network modifier, even when replacing a former oxide, such as SiO_2_. *In vitro* bioactivity assays in Kokubo SBF indicated a delay in the formation of bone-like apatite with increasing SrO concentration. Specifically, apatite formation was observed from 8 h of immersion for the SrO-free composition (X = 0 mol %), while in SrO-containing samples (X > 0 mol %), precipitation was detected only after 24 h. Dissolution tests in minimum essential medium (MEM) showed increased release of Sr^2+^ and Ca^2+^ ions with rising SrO content and as a function of soaking time, along with fluctuations in P^5+^ ion concentrations. These trends could suggest several variations in the dissolution rate associated with SrO incorporation. However, since no data was provided regarding the release of Si^4+^ ions, such hypotheses should be carefully addressed to discuss, from an accurate point of view, the bioactive behavior of the materials. The synthesized BGs were embedded in poly­(vinyl alcohol) (PVA) scaffolds.

**Table 5:** Summary of Designed BG Compositions, Where SrO Is Incorporated by Replacing Other Oxides than CaO

glass composition (mol %)						in vitro bioactivity			cell assays		bacterial assays		
SiO_2_	CaO	P_2_O_5_	SrO	other oxides	structural features	solution	immersion times	bioactive phases	cell line	significant results	bacterial strain	significant results	ref
85	10	5	0		amorphous	Kokubo’s SBF	8, 24, and 72 h	bone-like apatite formation after 8 h of soaking	not reported		not reported		^96^
82.5	10	5	2.5		amorphous			bone-like apatite formation after 24 h of soaking					
80	10	5	5		amorphous			bone-like apatite formation after 24 h of soaking					
36.9	24.76	3.88	0	34.44 (MgO)	amorphous	Kokubo’s SBF	3, 8, and 15 days	bone-like apatite formation after 15 days of soaking	not reported		not reported		^97^
36.9	24.76	3.88	1.64	32.8 (MgO)	amorphous			delayed bone like apatite formation after 15 days of soaking					
36.9	24.76	3.88	2.78	31.66 (MgO)	amorphous			superior bone-like apatite formation after 15 days of soaking					
46.14	26.91	2.6	0	24.35 (Na_2_O)	amorphous	not reported			not reported		not reported		^98^
48.68	19.12	2.75	3.76	25.69 (Na_2_O)	amorphous								
49.11	14.61	2.77	7.59	25.92 (Na_2_O)	amorphous								
46.14	26.91	2.6	0	24.35 (Na_2_O)	crystalline phases	Kokubo’s SBF	7 and 21 days	bone-like apatite formation since 7 days of soaking	NIH3T3 fibroblast cell line.	cell adhesion and viability	not reported		^99^
46.16	26.82	2.6	0.06	24.36 (Na_2_O)									
46.18	26.72	2.61	0.12	24.37 (Na_2_O)									
46.15	26.59	2.6	0.3	24.36 (Na_2_O)						decrease in cell adhesion and viability			
53.85	21.77	1.72	0	22.65 (Na_2_O)	crystalline phases	Kokubo’s SBF	24, 72, and 168 h	β-TCP, CaCO_3_, bone-like apatite formation	not reported		*S. aureus*, *E. coli*, *E. faecalis*, *S. mutans*, *C. tropicalis*, *C. glabrata*, *C. albicans*, *C. krusei*	antibacterial activity	^100^
54.53	19.29	1.74	1.49	22.94 (Na_2_O)	crystalline phases			β-TCP, CaCO_3_, bone-like apatite formation				antibacterial activity	
55.23	16.75	1.76	3.02	23.23 (Na_2_O)	crystalline phases			β-TCP, CaCO_3_, bone-like apatite formation				antibacterial activity	
46.1	26.9	2.6	0	24.4 (Na_2_O)	amorphous	Kokubo’s SBF	8 h, 7 and 14 days	superior bone-like apatite formation	cell line NCTC clone 929	cell viability	*E. coli*	antibacterial activity	^101–103^
44.1	26.9	2.6	0	24.4 (Na_2_O), 2 (Al_2_O_3_)	amorphous			bone-like apatite formation		cell viability		antibacterial activity	
45.2	26.4	2.5	2	23.9 (Na_2_O)	amorphous			superior bone-like apatite formation		cell viability		antibacterial activity	
43.2	26.4	2.5	2	24.4 (Na_2_O), 2 (Al_2_O_3_)	amorphous			bone-like apatite formation		cell viability		antibacterial activity	
52.42	20.22	3.11	1.22	22.87 (Na_2_O), 0.17 (Na_2_SiF_6_)	not reported	modified SBF	1, 7, 14, and 21 days	bone-like apatite formation after 7 days of soaking	not reported		*S. aureus* concentrations: 16, 32, 64, and 128 mg/mL	higher antibacterial effect	^104^
52.97	18.16	3.14	2.46	23.11 (Na_2_O), 0.17 (Na_2_SiF_6_)	not reported			bone-like apatite formation after 7 days of soaking				antibacterial effect	
53.53	16.06	3.17	3.72	23.35 (Na_2_O), 0.17 (Na_2_SiF_6_)	not reported			bone-like apatite formation after 7 days of soaking				antibacterial effect	
48.68	16.23	2.63	0	32. 46 (Na_2_O)	amorphous	not reported			not reported		not reported		^105^
47.12	15.71	2.69	3.07	31.41 (Na_2_O)	amorphous matrix with crystalline contributions								
46.46	36.27	1.07	0	6.6 (Na_2_O), 6.6 (K_2_O)	not reported	not reported			not reported		not reported		^106^
46.99	34.46	1.02	5	6.27 (Na_2_O), 6.27 (K_2_O)	not reported								
46.14	26.91	2.6	0	24.35 (Na_2_O)	amorphous	not reported			not reported		not reported		^107^
47.05	26.32	2.66	2.42	20.78 (Na_2_O), 0.77 (ZnO)	amorphous								
68.77	26.31	4.91	0		amorphous and crystalline contributions	Kokubo’s SBF	21 days	bone-like apatite formation	not reported		not reported		^108^
69.44	24.45	4.96	1.15										
46.1	26.91	2.6	0	24.35 (Na_2_O)	amorphous	Kokubo’s SBF	12, 24, 48, 96, 336 y 672 h	bone-like apatite formation after 96 h of soaking	human osteosarcoma Saos-2 cell: 100, 50, 25, 12.5 mg/mL	cell viability up to 12.5 mg/mL	*E. coli*, *S. aureus*, *S. mutans*	antibacterial activity	^109^
45.64	26.64	2.57	1	24.11 (Na_2_O)	amorphous			bone-like apatite formation after 24 h of soaking		cell viability up to 25 mg/mL		antibacterial activity	
45.18	26.37	2.55	2	23.86 (Na_2_O)	amorphous			bone-like apatite formation after 24 h of soaking		cell viability up to 25 mg/mL		antibacterial activity	

Kaur et al. (2021)^97^ synthesized BGs by the melt-quenching method, replacing MgO with SrO in the composition 36.92SiO_2_-3.88P_2_O_5_-(34.44–X)­MgO-24.76CaO-XSrO, where X = 0, 1.64, and 2.78 mol %. According to their findings, the incorporation of SrO led to variations in the glass transition temperature (*T*_g_), crystallization temperatures (*T*_c1_ and *T*_c2_), and microhardness values. Specifically, *T*_g_ values were 587 °C, 578 °C, and 609 °C; *T*_c1_ values were 621 °C, 665 °C, and 623 °C; and *T*_c2_ values were 768 °C, 719 °C, and 772 °C for samples with X = 0, 1.64, and 2.78 mol % SrO (Figure 7). A corresponding increase in microhardness was observed: 4.89, 4.86, and 5.54 GPa for samples with X = 0, 1.64, and 2.78 mol % SrO, respectively. In contrast, density values decrease notably: 4.01 g/cm^3^, 3.10 g/cm^3^, and 3.14 g/cm^3^, respectively. This simultaneous increase in hardness and decrease in density appear contradictory, as higher hardness is generally expected to correlate with increased or stable density. The authors suggested that these variations could be attributed to changes in the nonbridging oxygen (NBO) content of the glass network due to the incorporation of SrO, although no quantitative data related with *Q*_Si_^*n*^ units was provided in this study. This highlights a limitation of the study and leaves the reported hardness–density relationship difficult to rationalize without further structural evidence. Ion release graphs were presented, only showing the ionic concentrations after 15 days of soaking in Kokubo SBF. The base composition (X = 0 mol %) presented a higher release of Si^4+^ Ca^2+^, Mg^2+^, and P^5+^ species compared to the other samples. However, with increasing SrO content in the samples, a reduction in Si^4+^ and P^5+^ concentrations together with an increment in Sr^2+^ concentrations was evidenced, while no significant differences in Ca^2+^ and Mg^2+^ species were observed. Nevertheless, the lack of ionic release data for different soaking times during the test limited a deep understanding of ionic release profiles of the samples caused by substituting SrO for MgO. Regarding bioactivity, bone-like apatite formation was observed in all samples after 15 days of immersion. However, the sample with X = 2.78 mol % showed a greater precipitation of this bioactive phase compared to the others, which could be linked to the variations in ionic concentrations. Nevertheless, the observations reported could not be correlated with the dissolution profiles of the samples due to the lack of information in this study. Furthermore, the compositions were tested for vancomycin (VANCO) loading. The results indicated an increase in VANCO loading efficiency with higher SrO content in the samples, although no detailed discussion on the loading mechanism was provided.

Some studies have explored the development of BGs with SrO, either as a replacement for multiple oxides or as an interstitial component within the glass network. In this context, such compositions are commonly referred to as Sr-doped BGs. For example, Sasaki and collaborators (2019)^98^ synthesized BGs using the melt-quenching technique, starting from the 45S5 composition (46.14SiO_2_-2.6P_2_O_5_-24.35Na_2_O-26.91CaO mol %). They proposed the two alternative compositions with the incorporation of SrO: (i) 48.68SiO_2_-2.75P_2_O_5_-25.69Na_2_O-19.12CaO-3.76SrO mol % and (ii) 49.11SiO_2_-2.77P_2_O_5_-25.92Na_2_O-14.61CaO-7.59SrO mol %. According to the authors, Sr^2+^ mainly replaces Ca^2+^ in the glass network. After the synthesis, amorphous particles with homogeneous sizes ranging from 5 to 8 μm were obtained. Dissolution tests carried out on the obtained powder in distilled water revealed an increase in the concentration of Si^4+^ and Sr^2+^ ions with a higher SrO content. For the Ca^2+^ ions, different trends were observed. In the initial 45S5 composition, a higher ionic concentration was detected on the first day of the test, followed by a decrease in concentration until day 21. For the samples with 3.76 and 7.59 mol % SrO, a similar trend was observed, with ionic concentrations initially decreasing from day 1 to day 3, followed by an increase in release until day 21, with the highest concentration occurring in the sample with 3.76 mol % SrO. However, no data was provided for the release of P^5+^ and Na^+^ ions. The BGs were incorporated into glass ionomer cement formulations.

Amudha et al. (2020)^99^ prepared mesoporous particles Sr-doped BGs, based on compositions derived from 45S5 (46.14SiO_2_–2.60P_2_O_5_–24.35Na_2_O–26.91CaO mol %). The compositions were as follows: 46.16SiO_2_-2.60P_2_O_5_-24.36Na_2_O-26.82CaO-0.06SrO; 46.18SiO_2_-2.61P_2_O_5_-24.37Na_2_O-26.72CaO-0.12SrO; and 46.15SiO_2_-2.60P_2_O_5_-24.36Na_2_O-26.59CaO–0.30SrO mol %. These samples were synthesized via the sol–gel method, followed by thermal treatment at 800 °C for 10 h. In all of the synthesized samples, the predominant presence of crystalline phases was observed, specifically sodium calcium phosphate silicate (Na_0.11_Ca_0.89_)­(P_0.11_Si_0.89_O_3_) and sodium calcium silicate (Na_2_Ca_2_Si_3_O_9_). The intensity of the crystalline peaks of these phases increased with the SrO concentration. However, no evidence of amorphous contributions characteristic of the glassy network of a BG was observed, nor were quantitative values of the phase content reported. The compressive strength was measured as 1.5 ± 0.3, 1.2 ± 0.4, 8.1 ± 0.5, and 11.2 ± 0.5 MPa for samples with 0, 0.06, 0.12, and 0.30 mol % SrO, respectively. These changes in compressive strength could be attributed to the crystalline phases present in the samples, instead of the SrO presence, since its content was below 0.5 mol %. However, no correlation between the structural and mechanical features can be proposed without quantitative data about crystalline phases. Additionally, variations in surface area and pore sizes were reported: surface areas were 8.77 ± 0.19, 6.84 ± 0.24, 18.2 ± 0.17, and 17.2 ± 0.13 m^2^/g, and pore sizes were 22.14 ± 0.34, 16.24 ± 0.24, 20.73 ± 0.27, and 14.09 ± 0.41 nm for samples with 0, 0.06, 0.12, and 0.30 mol % SrO, respectively. In drug loading tests (using amoxicillin), improvements in encapsulation efficiency were noted: 88%, 89%, 92%, and 90%, and in drug loading efficiency: 38%, 39.5%, 41.2%, and 40.2% for samples with 0, 0.06, 0.12, and 0.30 mol % SrO, respectively. A decrease in drug release efficiency was observed: 85%, 58%, and 75% for the samples with 0, 0.06, 0.12, and 0.30 mol % SrO, respectively, compared to the initial composition, which had a release efficiency of 96%. This difference was likely due to variations in the porosity and surface area of the samples. In addition, all samples exhibited high hemocompatibility, with hemolysis effects <5% after *in vitro* test. Bone-like apatite formation was observed in all samples after 7 days of immersion in Kokubo SBF. However, cellular tests with NIH3T3 fibroblasts revealed that the sample with the highest SrO content (0.30 mol %) promoted adverse effects, including reduced cell adhesion and viability compared to the other samples evaluated. These effects were attributed to the material’s porosity and SrO content. No ionic release data for these compositions were provided in the study.

Maciel et al. (2020)^100^ fabricated BGs with varying concentrations of SrO in the formulations based on the 53.85SiO_2_-22.65Na_2_O-1.72P_2_O_5_-21.77CaO mol % composition. The BGs prepared were as follows: (i) 54.53SiO_2_-22.94Na_2_O-1.74P_2_O_5_-19.29CaO-1.49SrO mol % and (ii) 55.23SiO_2_-23.23Na_2_O-1.76P_2_O_5_-16.75CaO-3.02SrO mol %. These Sr-doped BGs were synthesized via the sol–gel method, followed by thermal treatment at 800 °C for 5 h. All the samples exhibited crystalline phases that were dependent on the SrO content. Initially, the formation of sodium–calcium-silicate phases such as Na_2_CaSi_2_O_6_ and Na_2_Ca_2_Si_3_O_9_ was observed in all of the samples. After the incorporation of SrO, additional phases, such as strontium silicate (SrSiO_3_) and phyllo-disilicate (Na_2_SrSi_2_O_6_), were formed. Ionic release tests in distilled water revealed an increase in Sr^2+^ ion concentration up to 6 h of immersion, followed by a reduction in release up to 12 h. At 12 h, the sample with the highest SrO content exhibited a greater release of Sr^2+^ ions. However, no data on the ionic concentrations of other elements present in the samples were provided. In addition, crystalline phases were observed in the samples along with the precipitation of bone-like apatite, β-Tricalcium phosphate (β-TCP), and calcite (CaCO_3_) after 168 h of immersion in Kokubo SBF. Additionally, all compositions demonstrated antibacterial effects against a range of microorganisms, including *S. aureus*, *E. coli*, *E. faecalis*, *S. mutans*, *C. tropicalis*, *C. glabrata*, *C. albicans*, and *C. krusei*. These effects were attributed to changes in local pH and the action of ions released from the samples. Finally, *in vivo* tests in Wistar rats were conducted for 14 and 28 days (3 groups with *n* = 5 for each period). The results showed that all of the samples promoted bone regeneration processes. However, the composition with 3.02 mol % SrO resulted in greater bone tissue formation and vascularization, which was attributed to the higher concentration of Sr^2+^ ions released from this sample. It was also noted that the BG samples were completely reabsorbed after 14 days of the test.

Araujo et al. (2020)^101^ proposed BG formulations based on the 45S5 composition (46.1SiO_2_-2.6P_2_O_5_-24.4Na_2_O-26.9CaO mol %). These doped BGs were: (i) 44.1SiO_2_-2.6P_2_O_5_-24.4Na_2_O-26.9CaO-2Al_2_O_3_ mol %, (ii) 45.2SiO_2_-2.5P_2_O_5_-23.9Na_2_O-26.4CaO–2SrO mol %, and (iii) 43.2SiO_2_-2.5P_2_O_5_-23.9Na_2_O-26.4CaO-2Al_2_O_3_-2SrO mol % and were obtained using the melt-quenching technique. Their results showed variations in glass transition temperatures (*T*_g_): 520 °C, 528 °C, 539 °C, and 530 °C, shown in Figure 7; differences in thermal stability windows (Δ*T*): 106 °C, 192 °C, 82 °C, and 171 °C; and changes in glass stability (KH): 0.19, 0.37, 0.16, and 0.38, for 45S5, BG with Al_2_O_3_, BG with SrO, and BG with Al_2_O_3_ + SrO, respectively. These findings suggest that the simultaneous addition of Al_2_O_3_ and SrO enhances glass stability, associated with a significant increase in the crystallization temperature and a broader processing window compared to the original 45S5 composition (Figure 7). Structural changes in the glassy network were also identified through analysis of *Q*_Si_^*n*^ units, showing (i) a decrease in the contributions from *Q*_Si_^4^ (3.31, 2.21, 1.97, 1.25%) and *Q*_Si_^3^ (39.20, 21.05, 18.59, 20.50%); (ii) an increase in *Q*_Si_^2^ (55.85, 73.08, 76.53, 77.49%); and (iii) variations in *Q*_Si_^0^ (1.63, 3.65, 2.91, 0.75%), for the respective compositions. These results indicate that upon incorporation into the glassy matrix, SrO tends to form aggregates with phosphate ions rather than contributing to the formation of a linear silicate network. However, the combined addition of SrO and Al_2_O_3_ appears to mitigate this effect, promoting a more linear glassy structure.

Later, Araujo et al. (2020)^102^ assessed the mechanical properties of the BG compositions previously reported in.^101^ Increases were observed in density (2.6920, 2.6948, 2.7108, and 2.7661 g/cm^3^) and Vickers hardness (3.6 ± 0.3, 3.8 ± 0.3, 4.4 ± 0.2, and 4.7 ± 0.3 GPa). Variations in elastic modulus were also reported (50 ± 1, 48 ± 2, 51 ± 2, 50 ± 2 GPa), along with differences in the coefficient of friction (2.1, 2.2, 2.1, 0.8) and wear rate (5.4 × 10^–5^, 5.4 × 10^–5^, 5.2 × 10^–5^, 2.2 × 10^–5^ mm^3^/Nm), for 45S5, BG with Al_2_O_3_, BG with SrO, and BG with Al_2_O_3_ + SrO, respectively. These changes were attributed to the effects induced by the incorporation of Al_2_O_3_ and SrO into the glass network. Subsequently, Araujo et al. (2021)^103^ evaluated the *in vitro* performance of these BG compositions.^101^ Bioactivity tests in Kokubo’s SBF revealed the formation of bone-like apatite in the BG containing Al_2_O_3_ + SrO as early as 8 h of immersion, and greater apatite precipitation in samples without Al_2_O_3_ after 14 days. Additionally, all compositions exhibited antibacterial activity against *E. coli*, although the BGs containing Al_2_O_3_ required a higher concentration (8 ppm) to achieve the same effect as the unmodified 45S5 and SrO-only samples (4 ppm). Finally, cell viability tests showed that extracts from all compositions at various concentrations (100, 50, 25, 12.5, and 6.25%) promoted the cellular viability. These effects were attributed to the ionic species released by the glass compositions. However, no information was provided regarding the dissolution behavior of the samples.

Li et al. (2022)^104^ synthesized BGs by the melt-quenching method using a base composition of 51.89SiO_2_-3.07P_2_O_5_-22.63Na_2_O-0.17Na_2_SiF_6_-22.24CaO mol %. They modified this composition by incorporating different concentrations of: (i) SrO: 52.42SiO_2_-3.11P_2_O_5_-22.87Na_2_O-0.17Na_2_SiF_6_-20.22CaO-1.22SrO mol %, 52.97SiO_2_-3.14P_2_O_5_-23.11Na_2_O-0.17Na_2_SiF_6_-18.16CaO-2.46SrO mol %, 53.53SiO_2_-3.17P_2_O_5_-23.35Na_2_O-0.17Na_2_SiF_6_-16.06CaO-3.72SrO mol %; (ii) ZnO: 52.25SiO_2_-3.10P_2_O_5_-22.79Na_2_O-0.17Na_2_SiF_6_-20.15CaO-1.54ZnO mol %, 52.62SiO_2_-3.12P_2_O_5_-22.95Na_2_O-0.17Na_2_SiF_6_-18.04CaO-3.11ZnO mol %, 52.99SiO_2_-3.14P_2_O_5_-23.11Na_2_O-0.17Na_2_SiF_6_-15.90CaO-4.69ZnO mol %. The *in vitro* assays demonstrated the precipitation of bone-like apatite after 7 days of immersion in Modified SBF (MSBF) for Sr-doped BGs, with increased apatite formation observed up to 21 days. In contrast, Zn-doped BGs exhibited concentration-dependent inhibition of apatite formation. For instance, in the sample with 1.54 mol % ZnO, apatite formation was evident only after 14 days, while in the 3.11 mol % ZnO composition, it appeared after 21 days. No apatite formation was observed for the highest ZnO concentration (4.69 mol %) even after 21 days. Based on these results, SrO-doped samples were selected for dissolution studies in MSBF. The release of Si^4+^ ions was highest in the 1.22 and 3.72 mol % SrO samples at days 1 and 7, although by days 14 and 21, ionic concentrations were comparable across all samples. A general increase in ion release was observed over time. For Ca^2+^, a decreasing trend in the concentration over time was noted. Interestingly, the 2.46 mol % Sr-doped BGs showed the highest Ca^2+^ concentration at 1 and 7 days, with all compositions exhibiting similar concentrations thereafter. Similarly, the P^5+^ ion concentration decreased over time. At 1 and 7 days, the highest release was observed in the 2.46 mol % Sr-doped BGs, while at later stages, the 3.72 mol % composition released more P^5+^. However, the study did not report data for Na^+^ or Sr^2+^ concentrations. Antibacterial activity tests were performed using material suspensions at 16, 32, 64, and 128 mg/mL. All samples inhibited *S. aureus* growth at 128 mg/mL, but the sample with 1.22 mol % SrO demonstrated superior inhibition at 32, 64, and 128 mg/mL. This effect was attributed to the combined release of ions and possible local pH variations.

Acevedo et al. (2021)^105^ developed BGs based on the composition 48.68SiO_2_-16.23CaO-32.46Na_2_O-2.63P_2_O_5_-XSrO-YK_2_O mol %. These compositions were: (i) 47.12SiO_2_-15.71CaO–31.41Na_2_O-2.69P_2_O_5_-3.07SrO mol %, (ii) 46.98SiO_2_-15.66CaO-31.32Na_2_O-2.68P_2_O_5_-3.36K_2_O mol % and were synthesized by melt-quenching. Vibrational spectroscopy confirmed the presence of Si–O, nonbridging oxygen (NBO), and PO_4_^3–^ bonds characteristic of the BG network. While predominantly amorphous structures were observed, both SrO- and K_2_O- doped samples showed minor crystalline peaks corresponding to sodium–calcium silicate (Na_2_CaSi_2_O_6_) and sodium calcium phosphate (NaCaPO_4_), suggesting the presence of crystalline domains embedded in an amorphous matrix. The effect of these BGs on dentin permeability was compared to commercial references (Bioglass and Biosilicate) using 84 bovine tooth samples in *in vitro* tests. All formulations reduced dentin permeability, with SrO-doped BG exhibiting the most significant reduction. The authors hypothesized that this improvement could be related to both synergistic effects of ion release which are (i) dentin restoration, (ii) decreased solubility in acidic environments, and (iii) potential enamel remineralization. However, no ion release studies were provided to support this hypothesis.

Barbeck and co-workers (2022)^106^ studied the immune response induced by BGs obtained from the composition ICIE16:46.46SiO_2_-6.6Na_2_O-36.27CaO-1.07P_2_O_5_-6.6K_2_O mol %. From this BG, two compositions were formulated: one with 5 mol % CuO (46.99SiO_2_-6.27Na_2_O-34.46CaO-1.02P_2_O_5_-6.27K_2_O-5CuO mol %) and one with 5 mol % SrO (46.99SiO_2_-6.27Na_2_O-34.46CaO-1.02P_2_O_5_-6.27K_2_O-5SrO mol %), which were fabricated by melt-quenching. After ionic release tests in SBF Kokubo, an increase in the concentration of Cu^2+^ and Sr^2+^ ions was observed with increasing days of the test, with the concentration of Cu^2+^ ions being higher than that of Sr^2+^ in the case of the doped samples after 14 days of immersion. However, no ionic concentration data were presented for the other elements in the samples or the initial composition. Subsequently, after *in vivo* assays in Sprague–Dawley rats for 10 and 30 days (*n* = 4), no significant differences were observed between the swelling and vascularization effects induced by the samples with CuO and SrO compared to the ICIE16 composition and 45S5, even though it has been widely reported that Cu^2+^ and Sr^2+^ ions can induce these effects. Due to these results, the authors recommended performing these tests at longer study times to corroborate possible long-term induced responses.

On the other hand, Özel and co-workers (2023)^107^ prepared BGs in the compositions 46.14SiO_2_-24.35Na_2_O-26.91CaO-2.6P_2_O_5_ mol % and 47.05SiO_2_-20.78Na_2_O-26.32CaO-2.66P_2_O_5_-2.42SrO-0.77ZnO mol %, which were also fabricated by the melt-quenching method. As reported, the incorporation of oxides does not induce crystalline effects in the BG. Also, an increase in the specific surface area of the particles (0.58 to 0.64 m^2^/g) and a decrease in the density (2.78 to 2.73 g/cm^3^) were observed with the addition of the dopants. Likewise, an increase in the glassy lattice connectivity values (2.15 to 2.18) was presented by incorporating both dopants. This result suggests changes in the NBOs of the glassy lattice with the incorporation of these oxides. These compositions were incorporated into sodium hyaluronate polymeric matrices, which were evaluated on an *in vitro* scale.

Ciolek et al. (2023)^108^ prepared BG compositions by the sol–gel method, which were formulated from the composition 68.77SiO_2_-4.91P_2_O_5_-26.31CaO mol % adding: (i) SrO: 69.44SiO_2_-4.96P_2_O_5_-24.45CaO-1.15SrO mol % and (ii) ZnO: 69.23SiO_2_-4.94P_2_O_5_-24.31CaO-1.46ZnO mol %. The obtained materials were subjected to several heat treatment conditions: (a) 550 °C for 3 h; (b) 600 °C for 3 h; (c) 600 °C for 10 h; (d) 650 °C for 3 h; (e) 650 °C for 10 h; (f) 1050 °C for 5 h. *T*_c_ values of 960 °C, 975 °C, and 950 °C were reported for the initial composition, the BG with SrO and the BG with ZnO, respectively. These values are much higher than those reported for traditional compositions, as seen in Figure 7. However, no *T*_g_ values were reported. Also, it was evidenced that changes in the intensities of the vibrational bands, the formation of crystalline phases, and the crystallinity of the compositions were directly associated with the heat treatment temperature used. The presence of crystalline phases immersed in amorphous matrices was observed in all samples. Nevertheless, increased crystalline phase percentages were evident with increasing heat treatment temperature: 22–41 wt % in samples obtained at 550 °C and between 71.8–99.8 wt % for compositions treated at 1050 °C. In samples heat treated at 1050 °C for 5 h, the presence of Pseudowollastonite (α-CaSiO_3_), wollastonite (β-CaSiO_3_), and quartz (SiO_2_) was observed. While in the sample with ZnO, the formation of hardystonite (Ca_2_ZnSi_2_O_7_) and cristobalite (SiO_2_) was evident as additional crystalline phases. In ion release assays in deionized water, it was evident that the release of Ca^2+^ ions was conditioned by the addition of SrO or ZnO and by the heat treatment temperatures, with ranges between 3 and 6 mg/mL after 24 h of the assay. However, ionic concentrations of the other elements in the manufactured BGs were not reported. For *in vitro* assays, samples treated at 550 °C for 5 h and at 650 °C for 10 h were selected. In the bioactivity evaluation at Kokubo SBF, it was evident that the samples presented the formation of bone-like apatite after 21 days of immersion. This result suggests that the presence of crystalline phases in the samples does not inhibit the bioactive response of the materials, but could decrease the rate of apatite formation, as reported in the literature.

Gavinho et al. (2023)^109^ modified the composition of 45S5 (46.1SiO_2_-2.6P_2_O_5_-24.4Na_2_O-26.9CaO mol %), to obtain samples with X = 0, Y = 1 mol %, X = 1, Y = 0 mol % (45.64SiO_2_-2.57P_2_O_5_-24.11Na_2_O-26.64CaO-XSrO-YMgO mol %); X = 0, Y = 2 mol %, X = 2, Y = 0 mol % (45.64SiO_2_-2.57P_2_O_5_-24.11Na_2_O-26.64CaO-XSrO-YMgO mol %). These BGs were fabricated by melt-quenching. Their results reported values of *T*_g_: 559, 552, and 551 °C, *T*_c_: 728, 710, 719 °C, and *T*_m_: 1175, 1159, 1154 °C for the initial composition, the sample with 2 mol % SrO and 2 mol % MgO, respectively, which were lower than those of 45S5 according to Figure 7. Also, it was determined that the addition of dopants does not affect the amorphous nature of the glass network but could generate changes in the NBOs of this network. In the cellular tests with Saos-2, extracts of all the passivated and nonpassivated samples were used at concentrations of 12.5, 25, 50, and 100 mg/mL. In the extracts of the nonpassivated samples, cell viability greater than 80% was observed for the samples with SrO (1 and 2 mol %) and MgO (1 and 2 mol %) for the 12.5 and 25 mg/mL extracts. At the same time, it was present at only 12.5 mg/mL for the initial composition. In the case of the extracts of the passivated samples, cell viability greater than 80% was determined for the extracts of the samples with SrO (1 and 2 mol %) and with 2 mol % MgO for concentrations up to 50 mg/mL, while the initial composition and the sample with 1 mol % MgO exhibited viability for concentrations up to 25 mg/mL. After the evaluation of bioactivity in SBF Kokubo, the formation of bone-like apatite was observed in the samples with SrO and MgO from 24 h of immersion. In comparison, the initial composition was evident from 96 h of the test. This result suggests that incorporating oxides at the concentrations used could accelerate the bioactive response of the compositions. Finally, in the antibacterial assays, an increase in the inhibition zone was observed in the samples with SrO (1 and 2 mol %) and MgO (1 and 2 mol %) compared to the initial composition against *E. coli*, *S. mutans*, and *S. aureus.* It is proposed that these variations could be related to the combined effect of the ions released by the samples. However, no data was reported regarding the ionic release process of the compositions.

## 6 Perspectives and Conclusions

This Review provides a comprehensive comparison of the properties of BGs within the SiO_2_-CaO-P_2_O_5_-SrO system, as reported in selected publications, and offers a summarized overview of the key findings and characteristics related to this system, as schematically illustrated in Figure 8. The type of substitution in the base composition and the concentration of incorporated SrO appear to influence the glass structure, particularly affecting the balance between nonbridging oxygens (NBOs) and bridging oxygens (BOs) within the glass network. These structural modifications likely play an important role in determining the mechanical behavior and bioactivity of the glasses. Notably, only six of the reviewed articles address changes in mechanical properties, related to structural changes in the glass network due to a possible occupation of Sr^2+^ in interstitial positions inside the network, highlighting a significant gap and an opportunity to advance the state of the art, especially in understanding how substitution type and SrO content (partial or total) impact mechanical behavior.

**Figure 8:**
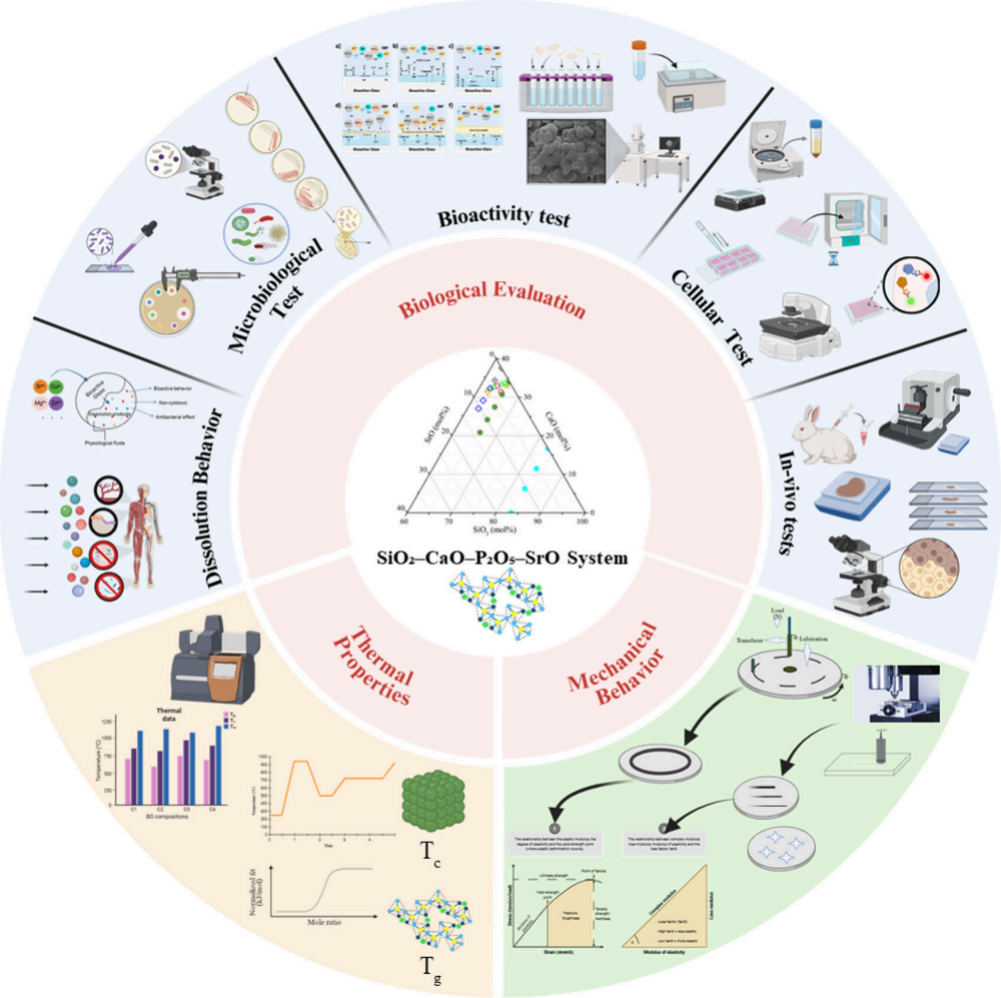
Overview of the key findings and characteristics related to the SiO_2_-CaO-P_2_O_5_-SrO system (created with Biorender: https://app.biorender.com/illustrations/682fb73db70a28a319cec705).

Most of the studies reviewed here have focused on the effects of substituting CaO with SrO in BG systems. While this approach has been thoroughly explored, replacing other oxides with SrO could also offer a valuable strategy for tailoring the properties of BGs. Interestingly, this Review found that very few studies have investigated this alternative. Among the few that do, the compositions (85–X)­SiO_2_-10CaO-5P_2_O_5_-XSrO mol %^96^ and 36.92SiO_2_-3.88P_2_O_5_-(34.44–X)­MgO-24.76CaO-XSrO mol %^97^ are particularly noteworthy. There are also some reports in which all oxide components in the composition are modified to include SrO. Among the papers explored in this review, only BG compositions within the SiO_2_-CaO-P_2_O_5_-SrO system from 14 works were presented in the ternary diagrams of Figure 6, considering a fixed value of P_2_O_5_ mol % and another oxides concentration up to 5 mol %. Interestingly, in both diagrams, there are zones of interest related to SrO content from 0–10 mol %, where most compositions are formulated. However, compositions with SrO content >16 mol % have not been explored yet. Thus, significant contributions to the state of the art are still required in this area, opening a wide window to explore.

Regarding the thermal characteristics summarized in Figure 7, there is an ongoing debate. Depending on (i) the type and quantity of oxides in the BG composition, (ii) the substitution strategy for incorporating SrO, and (iii) the amount of SrO added, thermal parameters such as *T*_g_, *T*_c_, and *T*_m_ may increase or decrease. These thermal changes are closely linked to devitrification processes and the nucleation of crystalline phases. This aspect is critical for applications requiring exposure to elevated temperatures, typically above 600 °C, such as sintering for dense 2D or 3D structures or the use of BGs as feedstock in thermal spray coatings. Crystalline phase formation may significantly reduce the material’s reactivity and compromise its biological performance. Therefore, it is essential to identify and quantify any crystalline phases formed upon SrO incorporation and assess their biological behavior, particularly regarding dissolution, bioactive phase formation, cellular response, and antimicrobial activity. A better understanding of the *in vitro* performance could pave the way for future *in vivo* evaluations.

In this review, several studies using sol–gel synthesis followed by thermal treatment were identified, resulting in (i) amorphous matrices with crystalline contributions, (ii) crystalline matrices with amorphous content, and (iii) fully crystalline materials. In such cases, thermal treatment temperature emerged as a decisive factor; if it exceeds the *T*_g_ of the composition, it may trigger crystallization. Hence, for applications requiring fully amorphous materials, it is recommended to determine the *T*_g_ prior to thermal treatment in the sol–gel process.

In terms of the *in vitro* performance, various effects associated with the initial composition and SrO content have been reported. For dissolution behavior, factors such as substitution type, SrO concentration, particle sizes, porosity, the structural nature of the material (fully amorphous, amorphous matrix with crystalline contributions, crystalline matrix with amorphous contributions, and fully crystalline), and the nature and proportion of other oxides significantly influence the release of ions like Sr^2+^, Ca^2+^, P^5+^, and Si^4+^. As highlighted in this Review, having detailed ion release profiles is crucial to correlate with biological responses observed in both *in vitro* and *in vivo* studies, ensuring scientific accuracy.

In SBF-based *in vitro* bioactivity evaluations, bone-like apatite formation has been reported for compositions with 2–18 mol % SrO, as shown in Tables 1 and 5. However, across the discussed studies, the effect of SrO addition on bioactive phase formation seems to be context-dependent and can be influenced by the synthesis route selected to produce the materials, the glass composition and its structural nature (fully amorphous, amorphous matrix with crystalline contributions, crystalline matrix with amorphous contributions, and fully crystalline), the dissolution processes of the materials, and the conditions established for the experimental evaluations, among others. Nonetheless, in cases where CaO is partially substituted by SrO, effects vary depending on the substitution ratio. For instance, substitution up to 50% (Table 2) may accelerate or delay bone-like apatite formation depending on the initial composition. The appearance of phases such as calcite (CaCO_3_), calcium orthosilicate (Ca_2_SiO_4_), and strontium silicate (SrSiO_3_) as additional phases postimmersion in Kokubo’s SBF has also been documented, suggesting altered ionic interactions with the physiological medium, where a possible competitive effect for Ca^2+^ species could affect the precipitation of bone-like apatite and promote the formation of the above-mentioned phases under physiological conditions. While calcite (CaCO_3_) formation is well-documented in some BGs, there remains a lack of clarity regarding the mechanisms and biological relevance of calcium orthosilicate (Ca_2_SiO_4_) and strontium silicate (SrSiO_3_) phases. In cases of >50% CaO substitution by SrO (Table 3), different phenomena have been observed, including: (i) the formation of Sr-substituted apatite, as Sr^2+^ can occupy Ca^2+^ positions in the apatite lattice; (ii) the formation of pure strontium apatite; and (iii) the emergence of strontium carbonate (SrCO_3_) as a bioactive phase. However, the formation mechanisms and biological effects of these phases remain underexplored at both *in vitro* and *in vivo* levels, warranting further investigation to determine their suitability for clinical applications.

According to the reviewed studies, cellular responses were primarily influenced by the concentrations of released ions, such as Sr^2+^, Ca^2+^, P^5+^, and Si^4+^. These ions influenced the behavior of various cell lines, including MLO-Y4, BM-MSC, hDPSCs, L929, MG-63, G292, U2-OS, MC3T3-E1, RAW 264.7, Saos-2, WJ-MSCs, NCTC clone 929, PBMC, and RBCs. Other factors, such as particle size, surface area, and porosity, also played important roles in modulating cellular responses. Based on these findings, the selection process for BG compositions with potential use in medical devices has become more refined. Additionally, the synergistic effects of released ions have shown promising antibacterial activity against microorganisms such as *S. epidermidis*, *E. faecalis*, *C. albicans*, *S. aureus*, *E. coli*, MRSA, *Streptococcus* spp., *S. mutans*, *C. tropicalis*, *C. glabrata*, and *C. krusei*.

Among several studies evaluated in this review, a recurring pattern was observed in which the intermediate SrO substitution level behaved differently from both the initial and the highest substitution levels. This nonlinear trend suggests that Sr^2+^ incorporation into the glass network does not simply follow a monotonic relationship with concentration. The aforementioned behavior could be associated with different factors, which include rearrangements in the silicate network, the generation of nonbridging oxygens (NBOs) that do not follow a direct proportionality with the SrO content, or competitive substitution between Sr^2+^ and Ca^2+^ within the glass structure. Another possibility is that partial substitution destabilizes the glass network, whereas higher substitution levels restore connectivity through the formation of Sr–O bonds or Sr-containing phases. Nevertheless, because most of the reviewed studies lack detailed structural analyses (e.g., *Q*_Si_^*n*^ distribution or Sr^2+^ site occupancy), the underlying mechanisms cannot yet be clearly established. This highlights a critical gap in the literature, underscoring the need for systematic studies to resolve why intermediate SrO levels frequently produce distinct property profiles.

Finally, although three of the reviewed studies included *in vivo* evaluations, further research is needed to better understand the *in vivo* behavior of SrO-containing BGs. This would enrich the existing knowledge base and help assess their potential for use in osteoporotic-related medical devices. It is also worth noting that most current studies are limited to cell culture and small animal models, indicating that significant work remains before advancing to clinical trials and the large-scale production and commercialization of SrO-containing BGs.
